# Supplementary material for: A class of organic cages featuring twin cavities
Source: Nat Commun. 2021 Oct 21;12:6124. doi: 10.1038/s41467-021-26397-3 (PMC8531300; doi:10.1038/s41467-021-26397-3)
Supplement: Supplementary file 1 — Supplementary Information [file 41467_2021_26397_MOESM1_ESM.pdf]

Supplementary Information

A class of organic cages featuring twin cavities

Zhenyu Yang[1], Chunyang Yu[1], Junjie Ding[1], Lihua Chen[1], Huiyu Liu[2], Yangzhi Ye[2], Pan Li[1], Jiaolong Chen[1], Kim Jiayi Wu[3], Qiang-Yu Zhu[1], Yu-Quan Zhao[1], Xiaoning Liu[1], Xiaodong Zhuang[1], and Shaodong Zhang[1*]

[1] School of Chemistry and Chemical Engineering, Frontiers Science Center for Transformative Molecules, Shanghai Jiao Tong University, 800 Dongchuan Road, Shanghai 200240 (China)
[*]E-mail: sdzhang@sjtu.edu.cn

[2] School of Physical Science and Technology, ShanghaiTech University, 393 Huaxia Middle Road, Shanghai 200120 (China)

[3] School of Chemistry, the University of Edinburgh, W. Mains Road, Edinburgh EH9 3FJ (U.K.)

Table of Contents

1. Materials......3
2. Techniques......3
2.1 Solution nuclear magnetic resonance (NMR)......3
2.2 Solid-state nuclear magnetic resonance (ssNMR)......4
2.3 Fourier-Transform Infrared Spectroscopy (FT-IR)......4
2.4 Matrix-assisted laser desorption and ionization time-of-flight mass spectrometry (MALDI-TOF MS)......4
2.5 Single crystal X-ray diffraction (SC-XRD)......4
2.6 Powder X-ray diffraction (PXRD)......5
2.7 High performance liquid chromatography (HPLC)......5
3. Synthesis......6
3.1 Synthesis route of model molecule 1......6
3.2 Synthesis route of model compound 2......9
3.3 Synthesis of Cage-2......11
4. Conformation capturing with diphane formation......12
4.1 Conformer-1 capturing with *endo*-[1,2,4]diphane formation......12
4.2 Conformer-1 and 2 capturing with *endo*- and *exo*-[1,2,5]diphane formation.14
4.3 HPLC analysis of the distribution of the diphanes......15
5. Kinetic experiment and diphanes transformation......16
6. Theoretical calculations......18
6.1 DFT calculations......18
6.2 Molecular dynamics simulations......20
7. X-ray crystallography......24
8. Powder X-ray diffraction pattern......30
9. Proton conductivity......31
10. Solid-state NMR spectroscopy (ssNMR)......35
11. Spectra......36
11.1 Mass spectra......36
11.2 IR spectra......39
11.3 NMR spectra......42
12. References......55

1. Materials

Dimethyl terephthalate, tetrakis(triphenylphosphine)palladium, phenyl lithium, Scandium(III) trifluoromethanesulfonate, tris(2-aminoethyl)amine (TREN), aniline were purchased from Beijing J&K Chemical Co. Ltd. 2-Formylbenzeneboronic acid and tris(3-aminopropyl)amine were purchased from Beijing InnoChem Co. Ltd. Sodium tris(acetoxy)hydroborate was purchased from Shanghai Adamas-Beta Co. Ltd. The key intermediates of 1,4-phenylenebis(diphenylmethanol) **1a**, 4,4'-(1,4-phenylenebis(diphenylmethylene))dianiline **1b**, 1,4-ditritylbenzene **1c**, 1,4-bis(tris(4-bromophenyl)methyl)benzene **1d** were prepared according to the literature procedure.[1] Tris(4-bromophenyl)methanol **2a** was synthesized according to the literature.[2] 4-(Tris(4-bromophenyl)methyl)phenol **2b** was prepared according to the literature.[3] All other reagents were bought from commercial sources and used without any purification unless stated. Tetrahydrofuran was dried over sodium/benzophenone under nitrogen atmosphere before use. The reaction evolution was monitored by thin-layer chromatography (TLC) and flash column chromatography was performed on silica gel (200-300 mesh) with indicated eluent.

2. Techniques

2.1 Solution nuclear magnetic resonance (NMR)

^{1}H NMR and ^{13}C NMR spectra were recorded on a Bruker Advance Ⅲ HD (400/500 MHz) NMR spectrometer at room temperature. Proton and carbon chemical shift values (δ) and coupling constants (J) are reported in ppm and Hertz (Hz) respectively. The resonance multiplicity of the ^{1}H NMR spectra are denoted as “s” (single), “d” (doublet), “t” (triplet), “quint” (quintet), “m” (multiplet) and broad resonances are described as “br”. Residual protic solvent of $CDCl_3$ (^{1}H, δ = 7.26 ppm; ^{13}C, δ = 77.16 ppm), $(CD_3)_2SO$ (^{1}H, δ = 2.50 ppm; ^{13}C, δ = 39.52 ppm), CD_2Cl_2 (^{1}H, δ = 5.32 ppm; ^{13}C, δ = 53.84 ppm), CD_3OD (^{1}H, δ = 3.31 ppm; ^{13}C, δ = 49.00 ppm) were used as the internal standard in the ^{1}H and ^{13}C NMR spectra.

2.2 Solid-state nuclear magnetic resonance (ssNMR)

Solid-state ^1H MAS single-pulse and DQ/SQ spectra were recorded on a Bruker AVANCE NEO 600 MHz, 3.2 mm rotor, MAS of 15 MHz, recycle delay of 5 sec.

2.3 Fourier-Transform Infrared Spectroscopy (FT-IR)

Fourier-Transform Infrared Spectroscopy (FT-IR) spectra were recorded on a Perkin-Elmer Paragon 1000 spectrometer at frequencies ranging from 4000 to 500 cm^{-1} at room temperature. The signal intensity was denoted as “s” (strong), “m” (medium), “w” (weak) and “br” (broad).

2.4 Matrix-assisted laser desorption and ionization time-of-flight mass spectrometry (MALDI-TOF MS)

Matrix-assisted laser desorption and ionization time-of-flight mass spectrometry (MALDI-TOF MS) was performed on a solariX XR 7.0 T hybrid quadrupole-FTICR mass spectrometer equipped with an ESI/APCI/MALDI ion source (Bruker Daltonics, Bremen, Germany). The MS instrument was tuned and calibrated with ESI-L low concentration tuning mix (Agilent Technologies, Santa Clara, CA, USA) and sodium formate. The analytical sample was prepared by mixing the reaction mixture (2.0 mg/mL in $CHCl_3$) with the matrix solution (10 mg/mL DCTB in $CHCl_3$) in a v/v ratio of 1/5, and then loaded onto the MALDI plate. The sample on the plate was thoroughly dried prior to analysis. The mass spectra were analyzed using Compass Data Analysis 5.0 (Bruker).

2.5 Single crystal X-ray diffraction (SC-XRD)

The crystals were kept at 170.0 K during data collection. Using Olex2[4], the structures were solved with the SHELXT[5] structure solution program using Intrinsic Phasing and refined with the SHELXL[6] refinement package using Least Squares minimisation. Some solvent molecules were highly disordered and could not be reasonably located. The voids in the crystal lattice were treated with Platon squeeze program.

2.6 Powder X-ray diffraction (PXRD)

The detailed procedure of sample preparation for PXRD measurements are as follows: The as-synthesized bulk powder samples of diphanes were firstly dispersed in distilled THF solvent with a concentration at 30 mg/mL, followed by sonication until thorough dispersion. Additional TFA was then added until the mixture became clear, which was subsequently subjected to filtration with 0.45 μm PTFE springe filter to remove the undissolved residue. Slow evaporation of the settled solution yielded high-quality single crystals, which were then loaded into a quartz glass capillary with a diameter of 0.7 mm without mother liquor. PXRD data were collected at ambient temperature with a capillary setup.

2.7 High performance liquid chromatography (HPLC)

HPCL analysis were performed on a Shimadzu LC-20 AD instrument at room temperature using a Daicel Chiralcel IA column. The elution was employed within 60 min with 18% $CHCl_3$ in ethanol containing 0.5% diethylamine of total volume at a flow rate of 0.6 mL/min. The sample concentration was 5.0 mM in methanol, and the injection volume was 10 μL. The absorbance of *endo*-[1,2,4]diphane was monitored at 254 nm, *endo*-[1,2,5]diphane and *exo*-[1,2,5]diphane were monitored at 298 nm.

3. Synthesis

3.1 Synthesis route of model molecule 1

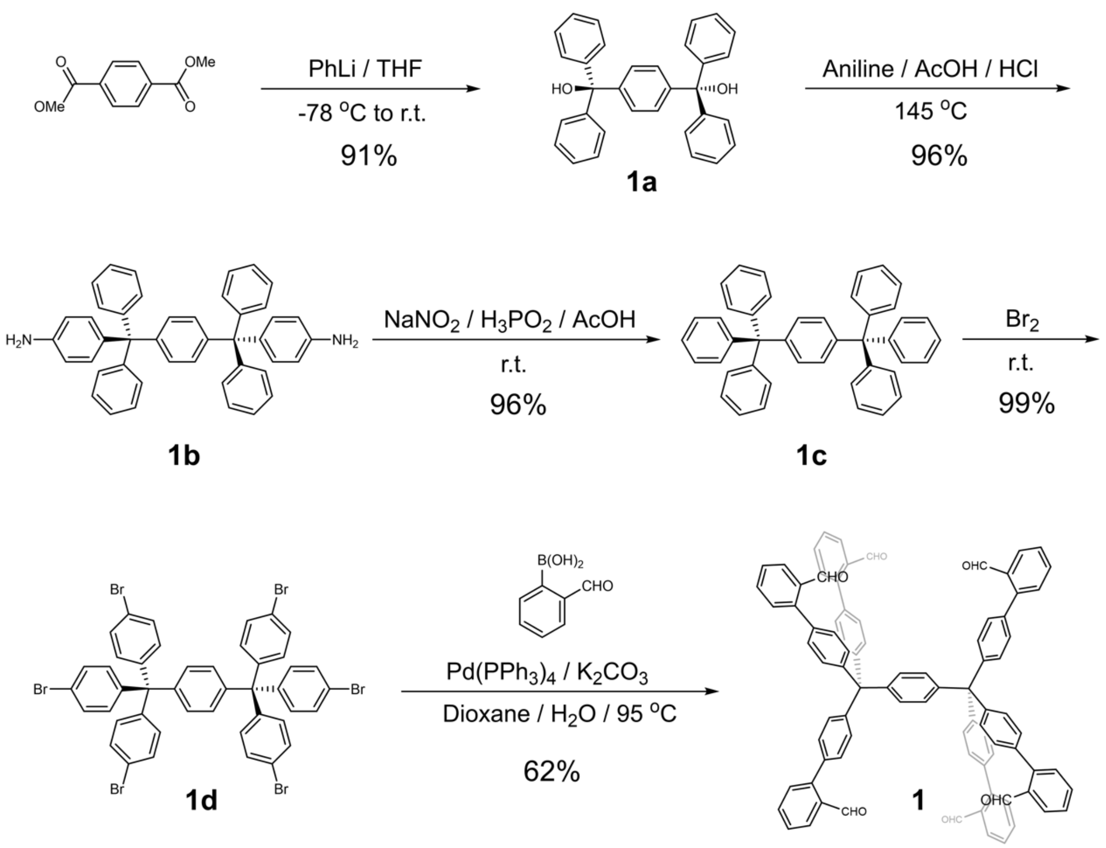

Supplementary Fig. 1 Synthesis route of model compound **1**.

1,4-Phenylenebis(diphenylmethanol) (1a): The phenyllithium solution (51.4 mL, 51.0 mmol, 5.0 equiv., 1.0 M in ether) in anhydrous THF (70.0 mL) was cooled to -78 °C under argon atmosphere. Into the mixture was added dropwise the solution of dimethyl terephthalate (2.0 g, 10.3 mmol, 1.0 equiv.) in anhydrous THF (25.0 mL). The reaction mixture was stirred for another 0.5 h at -78 °C and then allowed to warm to room temperature for 24 h. Then, the reaction was quenched with a sat. aqueous NH_4Cl solution (50.0 mL), and THF was removed by rotary evaporator. The reaction was extracted with DCM (3×40.0 mL), dried over anhydrous Na_2SO_4, and concentrated to give the crude product. Purification by flash column chromatography (Hex/DCM = 1:3 v/v, as eluent) afforded the title compound **1a** (4.1 g, 91%) as a white solid. ^{1}H NMR (400 MHz, $CDCl_3$), δ = 7.33 (m, 24H), 2.86 (s, 2H). ^{13}C NMR (101 MHz, $CDCl_3$), δ = 146.81, 145.88, 128.06, 128.00, 127.68, 127.41, 81.99.

4,4'-(1,4-Phenylenebis(diphenylmethylene))dianiline (1b): Into a 50 mL flask was charged with aniline (3.7 g, 39.9 mmol, 9.5 equiv.), conc. HCl (2.5 mL) and glacial acetic acid (20.0 mL), then 1,4-phenylenebis(diphenylmethanol) **1a** (1.9 g, 4.2 mmol, 1.0 equiv.) was added. The reaction mixture was refluxed at 140 °C for 12 h. After cooling to room temperature, the formed grey solid was filtered off under vacuum and washed with acetic acid (100.0 mL) and diethyl ether (100.0 mL). The solid was dissolved in methanol and precipitated in diethyl ether afforded the title compound **1b** (2.4 g, 96%) as a white solid. ^{1}H NMR (400 MHz, DMSO), δ = 7.26 (t, J = 12.0, 8.0 Hz, 8H), 7.17 (d, J = 8.0 Hz, 4H), 7.09 (d, J = 8.0 Hz, 8H), 7.0 (s, 4H), 6.72 (d, J = 8.0 Hz, 4H), 6.45 (d, J = 8.0 Hz, 4H), 5.05 (s, 4H). ^{13}C NMR (101 MHz, DMSO), δ = 147.07, 146.30, 144.36, 133.34, 131.07, 130.47, 129.56, 127.42, 125.71, 113.06, 63.27.

1,4-Ditritylbenzene (1c): Dianiline **1b** (3.0 g, 5.0 mmol, 1.0 equiv.) was added into glacial acetic acid (265.0 mL) and hypophosphoric acid (31.0 mL, 50% aqueous solution). Then, the reaction mixture was cooled to 0 °C, and $NaNO_2$ (1.0 g, 15.0 mmol, 3.0 equiv.) was added under vigorous stirring. The reaction mixture was stirred for 24 h at room temperature, and then water (50.0 mL) was added. The formed solid was filtered off under vacuum and washed with water, methanol, and diethyl ether (50.0 mL), respectively, and then dried under vacuum, yielding the title compound **1c** (2.7 g, 96%) as a beige solid. ^{1}H NMR (400 MHz, $CDCl_3$), δ = 7.23–7.24 (m, 12H), 7.18–7.20 (m, 18H), 7.07 (s, 4H). ^{13}C NMR (101 MHz, $CDCl_3$), δ = 146.85, 144.39, 131.36, 130.42, 127.50, 126.07, 64.72.

1,4-Bis(tris(4-bromophenyl)methyl)benzene (1d): Into a 50 mL flask was charged with 1,4-ditritylbenzene **1c** (3.5 g, 6.0 mmol, 1.0 equiv.) and cooled to -30 °C, followed by addition of pure bromine (5.7 mL, 108.0 mmol, 18.0 equiv.) under vigorous stirring. The reaction mixture was allowed to room temperature and stirred for another 1 h. The reaction was then cooled to -30 °C again, and ethanol (50.0 mL) was added and stirred for another 0.5 h at room temperature. The formed solid was filtered off under vacuum, washed with water (250.0 mL) and methanol (250.0 mL), and then dried under vacuum, yielding the title compound **1d** (6.2 g, 99%) as a light-yellow solid. ^{1}H NMR (400 MHz,

$CDCl_3$), δ = 7.38 (dd, J = 8.0, 4.0 Hz, 12H), 6.98–7.01 (m, 16H). ^{13}C NMR (101 MHz, $CDCl_3$), δ = 144.78, 143.74, 132.62, 131.08, 130.38, 120.85, 63.70.

Model molecule 1: Into a 100 mL Schlenk flask was charged with 1,4-bis(tris(4-bromophenyl)methyl)benzene **1d** (514.8 mg, 0.5 mmol, 1.0 equiv.), (2-formylphenyl)boronic acid (900.0 mg, 6.0 mmol, 12.0 equiv.), $Pd(PPh_3)_4$ (173.0 mg, 0.15 mmol, 0.3 equiv.), K_2CO_3 (2.0 g, 15.0 mmol, 30.0 equiv.), which was degassed by evacuating and refilling with nitrogen three times. After which, dioxane (40.0 mL) and water (10.0 mL) were added successively under N_2 atmosphere. The reaction mixture was refluxed at 95 ℃ for 2 days. After cooling to room temperature, dioxane was removed by rotary evaporator and the left mixture was extracted with DCM (3×50.0 mL), dried over anhydrous Na_2SO_4, and concentrated to give the crude product. Purification by flash column chromatography (pure DCM as eluent) yielded the title compound **1** (368.0 mg, 62%) as a white solid. ^{1}H NMR (400 MHz, $CDCl_3$), δ = 10.06 (s, 6H), 8.02 (d, J = 4.0 Hz, 6H), 7.65 (t, J = 12.0, 8.0 Hz, 6H), 7.49–7.52 (m, 12H), 7.41 (d, J = 8.0 Hz, 12H), 7.37 (d, J = 8.0 Hz, 12H), 7.31 (s, 4H). ^{13}C NMR (101 MHz, $CDCl_3$), δ =192.64, 146.32, 145.54, 144.34, 135.96, 133.83, 131.27, 130.91, 130.72, 129.73, 128.03, 127.80, 64.44. FT-IR (KBr, cm^{-1}): ν = 3358.1 (br), 3192.6 (w), 2954.4 (w), 2920.3 (s), 2850.4 (m), 1692.7 (s), 1658.8 (w), 1633.1 (w), 1596.1 (m), 1470.8 (m), 1391.3 (w), 1253.3 (m), 1192.8 (m), 1003.4 (w), 827.6 (m), 763.9 (m), 649.8 (w). HR-MS (MALDI): $C_{73}H_{49}O_5$ $(M)^+$, Calculated: 1005.3575; Found: 1005.3648, which corresponds to the cationic fragment of the title molecule with one arm, *i.e.*, two benzene units, stripped off by laser.

3.2 Synthesis route of model compound 2

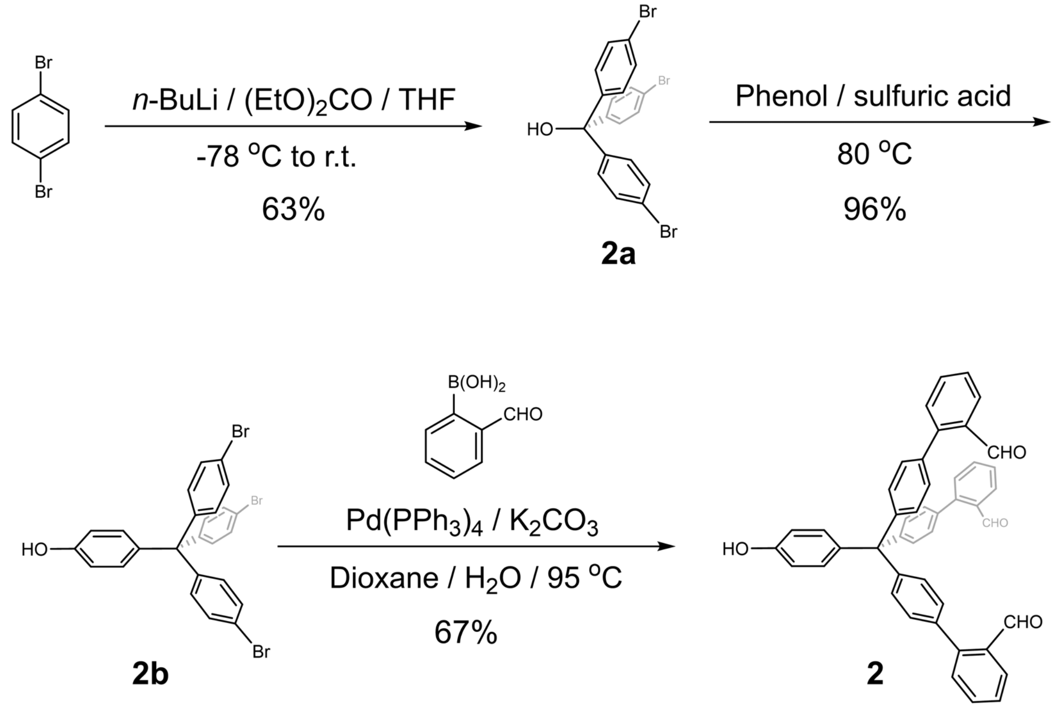

Supplementary Fig. 2 Synthesis route of model compound **2**.

Tris(4-bromophenyl)methanol (2a): 1,4-Dibromobenzene (11.8 g, 50.0 mmol, 3.0 equiv.) was dissolved in anhydrous THF (100.0 mL) and cooled to -78 ℃, followed by dropwise addition of *n*-BuLi (1.6 M in Hexane) (31.2 mL, 50.0 mmol, 3.0 equiv.). The mixture was stirred for 1 h, after which diethyl carbonate (2.0 g, 16.6 mmol, 1.0 equiv.) was added slowly. The solution was stirred for another 0.5 h at -78 ℃ and then allowed to room temperature for 12 h. Saturated NH_4Cl aqueous solution (50.0 mL) was added to quench the reaction, which was then extracted with DCM (3×50.0 mL), dried over anhydrous Na_2SO_4, and concentrated to give the crude product. Purification by flash column chromatography (Hex/DCM = 1:3, v/v) yielded the title compound **2a** (5.2 g, 63%) as a white solid. ^{1}H NMR (400 MHz, $CDCl_3$), δ = 7.45 (dd, J = 8.0, 4.0 Hz, 6H), 7.12 (dd, J = 8.0, 4.0 Hz, 6H), 2.70 (s, 1H). ^{13}C NMR (101 MHz, $CDCl_3$), δ = 144.99, 131.47, 129.60, 122.11, 81.24.

4-(Tris(4-bromophenyl)methyl)phenol (2b): Tris(4-bromophenyl)methanol **2a** (2.0 g, 4.0 mmol, 1.0 equiv.) and phenol (2.0 g, 21.9 mmol, 5.5 equiv.) were charged into a 25 mL Schlenk flask, which was degassed by evacuating and refilling with argon three

times. Sulfuric acid (250 μL) was then added, and the system was heated to 80 ℃ for 4 h under argon. After cooling to room temperature, an aqueous solution of NaOH (10 wt%, 25.0 mL) was added and stirred for 0.5 h to remove the excess phenol. The mixture was then extracted with DCM (3×50.0 mL), dried over anhydrous Na_2SO_4, and concentrated to give the pure product **2b** (2.2 g, 96%) as a white solid. ^{1}H NMR (400 MHz, $CDCl_3$), δ = 7.38–7.41 (dd, J = 8.0, 4.0 Hz, 6H), 7.04–7.07 (dd, J = 8.0, 4.0 Hz, 6H), 6.98–7.01 (dd, J = 8.0, 4.0 Hz, 2H), 6.72–6.75 (dd, J = 8.0, 4.0 Hz, 2H). ^{13}C NMR (101 MHz, $CDCl_3$), δ = 154.55, 145.73, 137.92, 132.93, 132.37, 131.20, 120.69, 115.01, 63.73.

Model molecule 2: Into a 250 mL Schlenk flask was charged with 4-(tris(4-bromophenyl)methyl)phenol **2b** (1.9 g, 3.3 mmol, 1.0 equiv.), (2-formylphenyl)boronic acid (2.9 g, 19.5 mmol, 6.0 equiv.), $Pd(PPh_3)_4$ (563.0 mg, 0.5 mmol, 0.15 equiv.), K_2CO_3 (6.7 g, 48.8 mmol, 15.0 equiv.), which was degassed by evacuating and refilling with nitrogen three times. Dioxane (130.0 mL) and water (32.5 mL) were then added successively under N_2 atmosphere. The reaction mixture was refluxed at 95 ℃ for 2 days. After cooling to room temperature, dioxane was removed by rotary evaporator and the left mixture was extracted with DCM (3×70.0 mL), dried over anhydrous Na_2SO_4, and concentrated to give the crude product. Purification by flash column chromatography (PE/EA = 1:1, v/v) yielded the title compound **2** (1.4 g, 67%) as a white solid. ^{1}H NMR (400 MHz, $CDCl_3$), δ = 10.06 (s, 3H), 8.02–8.04 (d, J = 8.0 Hz, 3H), 7.63–7.67 (t, J = 16.0, 8.0 Hz, 3H), 7.49–7.52 (m, 6H), 7.40 (d, J = 8.0 Hz, 6H), 7.34 (d, J = 8.0 Hz, 6H), 7.17 (d, J = 8.0 Hz, 2H), 6.83 (d, J = 8.0 Hz, 2H). ^{13}C NMR (101 MHz, $CDCl_3$), δ = 192.73, 154.21, 146.75, 145.66, 138.31, 135.72, 133.82, 133.80, 132.42, 131.15, 130.92, 129.68, 127.99, 127.77, 114.90, 64.21. FT-IR (KBr, cm^{-1}): ν = 3361.5 (br), 2956.4 (s), 2924.5 (s), 2852.4 (s), 1692.7 (s), 1596.4 (m), 1509.1 (w), 1493.0 (w), 1473.9 (m), 1378.1 (w), 1363.3 (w), 1254.9 (m), 1193.3 (m), 1081.6 (m), 1004.2 (w), 968.5 (m), 829.4 (s), 767.3 (s), 652.1 (m), 597.6 (m), 536.5 (w). HR-MS (MALDI): $C_{33}H_{23}O_3^+$ $(M)^+$, Calculated: 467.1642; Found: 467.1658, which

corresponds to the cationic fragment of the title molecule with one arm, *i.e.*, two benzene units, stripped off by laser.

3.3 Synthesis of Cage-2

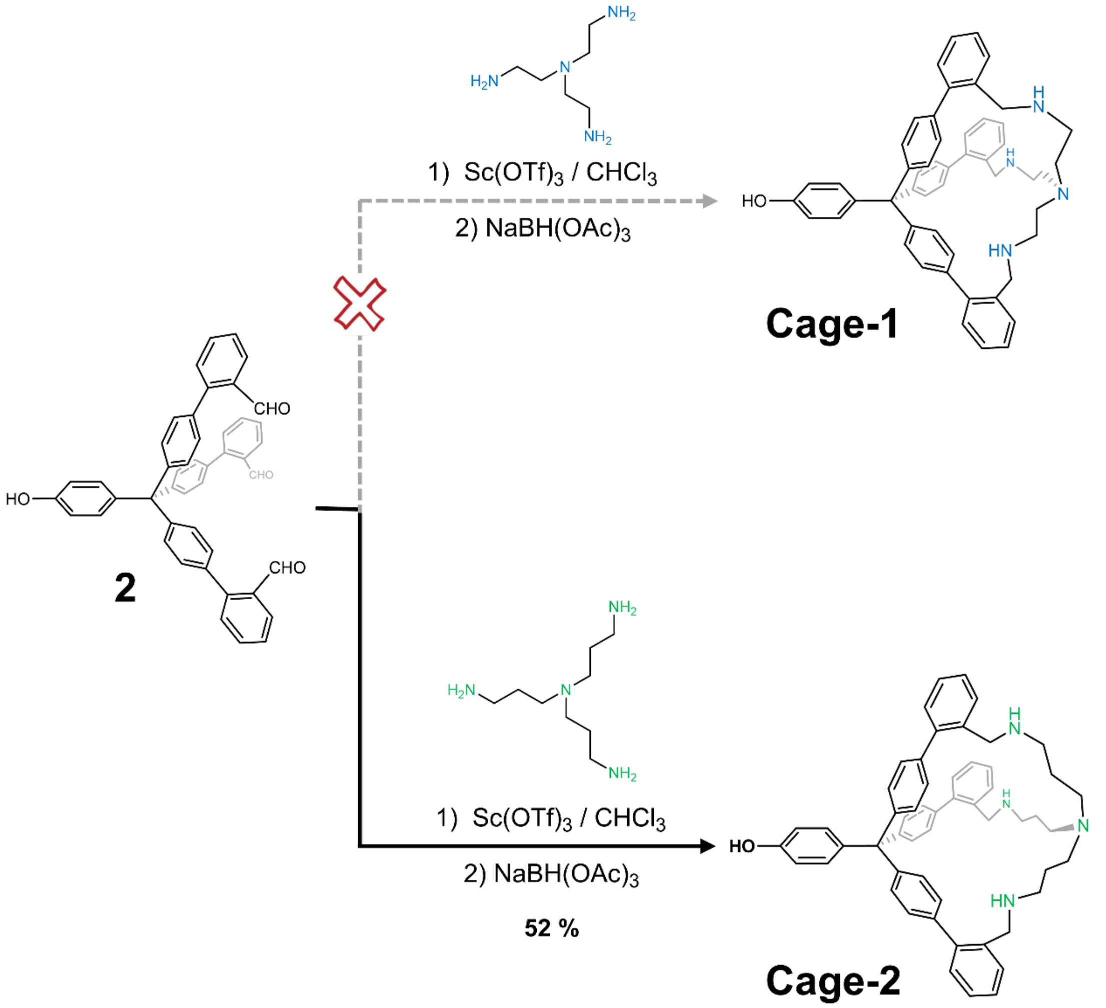

Supplementary Fig. 3 Synthesis of Cage-2.

Cage-2: Into a 500 mL flask was charged with 0.005 mol/L model molecule **2** (492.7 mg, 0.76 mmol, 1 equiv.) 152 mL in $CHCl_3$, then a 0.005 mol/L solution of tris(3-aminopropyl)amine (CC-2) (135.9 mg, 0.76 mmol, 1.0 equiv.) 228.0 mL in $CHCl_3$ was added dropwise followed by addition of $Sc(OTf)_3$ (113.0 mg, 0.3 mmol, 0.3 equiv.) directly. The reaction mixture was stirred at room temperature for 4 h. Then, the product was reduced by $NaBH(OAc)_3$ (1.2 g, 5.7 mmol, 7.5 equiv.) overnight, the excess reductant $NaBH(OAc)_3$ was filtered off under vacuum and the solution was quenched with NaOH solution (2.0 M, 200 mL), extracted with $CHCl_3$ (3×200 mL), dried over

anhydrous Na_2SO_4, and concentrated to give the crude product. Purification by flash column chromatography (DCM/MeOH/NH_3(aq) = 50:2:3, v/v/v) afforded the Cage-2 (312.0 mg, 52%) as a white solid. ^1H NMR (400 MHz, CD_2Cl_2), δ = 7.38 (d, J = 8.0 Hz, 6H), 7.26–7.32 (m, 18H), 7.20 (d, J = 8.0 Hz, 2H), 6.2 (d, J = 8.0 Hz, 2H), 7.36 (s, 6H), 2.43–2.47 (t, J = 16.0, 8.0 Hz, 6H), 2.26–2.30 (d, J = 16.0, 8.0 Hz, 6H), 1.27–1.41 (m, 6H). ^{13}C NMR (101 MHz, $CDCl_3$), δ = 155.27, 146.79, 142.18, 139.26, 138.12, 132.84, 131.43, 130.79, 130.04, 128.53, 127.89, 127.70, 114.99, 64.08, 52.74, 52.26, 47.71, 27.04. FT-IR (KBr, cm^{-1}): ν = 3024.2 (w), 2925.2 (s), 2852.8 (m), 1664.9 (w), 1609.2 (m), 1509.3 (s), 1481.0 (s), 1446.7 (m), 1376.7 (w), 1263.9 (s), 1178.3 (s), 1108.7 (m), 1006.1 (m), 828.4 (s), 762.1 (s), 737.3 (s), 597.0 (m), 570.5 (w), 537.6 (m). HR-MS (MALDI): $C_{55}H_{57}N_4O^+$ $(M+H)^+$, Calculated: 789.4527; Found: 789.4503.

4. Conformation capturing with diphane formation

4.1 Conformer-1 capturing with *endo*-[1,2,4]diphane formation

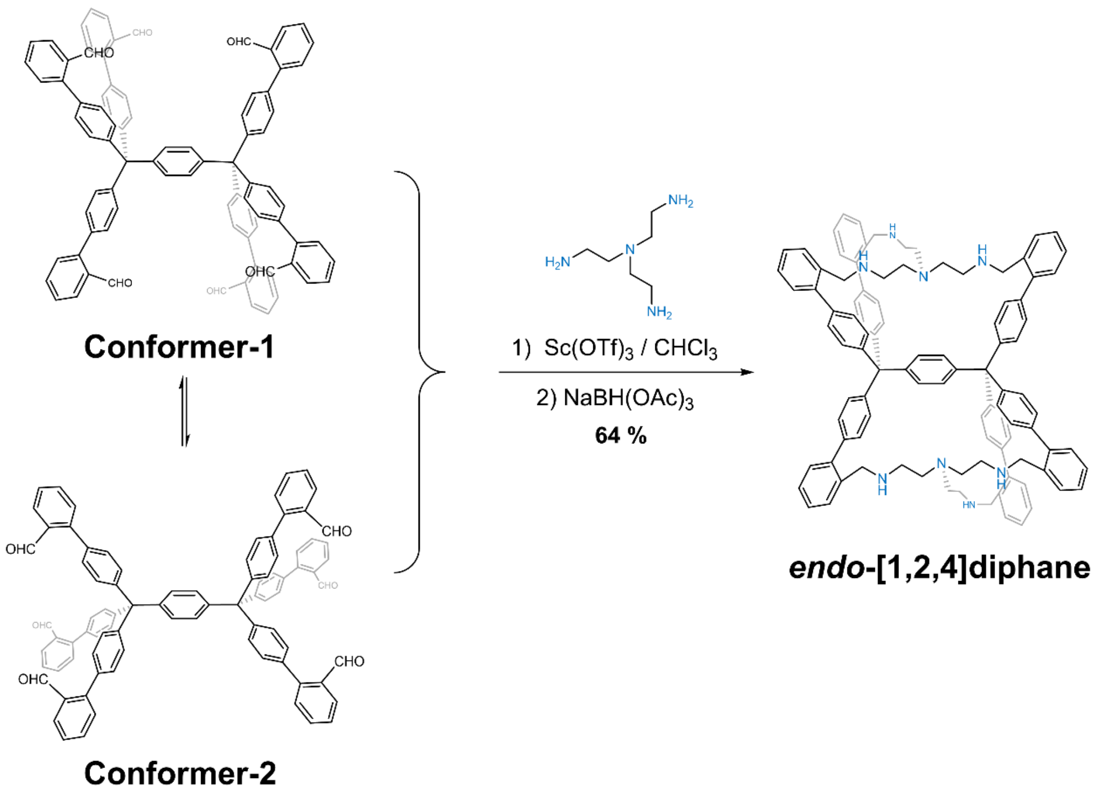

Supplementary Fig. 4 Selective Conformer-1 capturing with *endo*-[1,2,4]diphane formation.

***endo*-[1,2,4]Diphane:** Into a 1000 mL flask was charged with 0.005 mol/L model molecule **1** (1.0 g, 0.84 mmol, 1 equiv.) 170 mL in $CHCl_3$, then a 0.005 mol/L solution of tris(2-aminoethyl)amine (CC-1) (247 mg, 1.7 mmol, 2 equiv.) 500 mL in $CHCl_3$ was added dropwise followed by addition of $Sc(OTf)_3$ (248 mg, 0.5 mmol, 0.6 equiv.). The reaction mixture was stirred at room temperature for 4 h. Then, the product was reduced by $NaBH(OAc)_3$ (2.6 g, 12.6 mmol, 15 equiv.) overnight, the excess reductant $NaBH(OAc)_3$ was filtered off under vacuum, and the solution was quenched with NaOH solution (2 M, 200 mL), extracted with $CHCl_3$ (3×200 mL), dried over anhydrous Na_2SO_4, and concentrated to give the crude product. Purification by flash column chromatography (DCM/MeOH/NH_3(aq) = 50:2:3, v/v/v) afforded the *endo*-[1,2,4]diphane (736 mg, 64%) as a white solid. NMR spectroscopy was conducted with additional trifluoroacetic acid (TFA) for better solubility in MeOD. ^{1}H NMR (400 MHz, MeOD), δ = 7.19–7.60 (m, 44H), 4.17 (s, 4H), 4.12 (d, J = 8.0 Hz, 4H), 4.03 (d, J = 8.0 Hz, 4H), 3.06–3.10 (m, 4H), 2.91–2.95 (m, 8H), 2.78–2.82 (m, 4H), 2.61–2.69 (m, 4H), 2.46–2.54 (m, 4H). ^{13}C NMR (101 MHz, $CDCl_3$), δ = 161.34, 160.95, 160.59, 160.17, 159.69, 159.27, 158.85, 158.44, 147.14, 146.78, 146.51, 144.10, 143.46, 139.72, 139.61, 132.68, 132.20, 132.16, 131.80, 131.47, 131.31, 131.17, 130.90, 130.67, 130.37, 130.15, 129.96, 129.85, 129.70, 129.63, 120.29, 118.34, 117.46, 115.49, 114.64, 112.61, 111.81, 65.76, 55.13, 54.90, 54.68, 54.45, 54.22, 53.99, 53.77, 50.02, 46.62, 45.23. FT-IR (KBr, cm^{-1}): ν = 3356.8 (br), 3195.3 (w), 3024.3 (w), 2920.4 (s), 2849.9 (s), 2380.9 (br), 1927.9 (br), 1659.8 (s), 1632.5 (s), 1481.3 (s), 1202.1 (s), 1125.1 (s), 1006.5 (w), 823.3 (m), 763.1 (s), 721.6 (m), 582.2 (w). HR-MS (MALDI): $C_{98}H_{95}N_8$ $(M+H)^+$, Calculated: 1384.7708; Found: 1384.7883.

4.2 Conformer-1 and 2 capturing with *endo*- and *exo*-[1,2,5]diphane formation

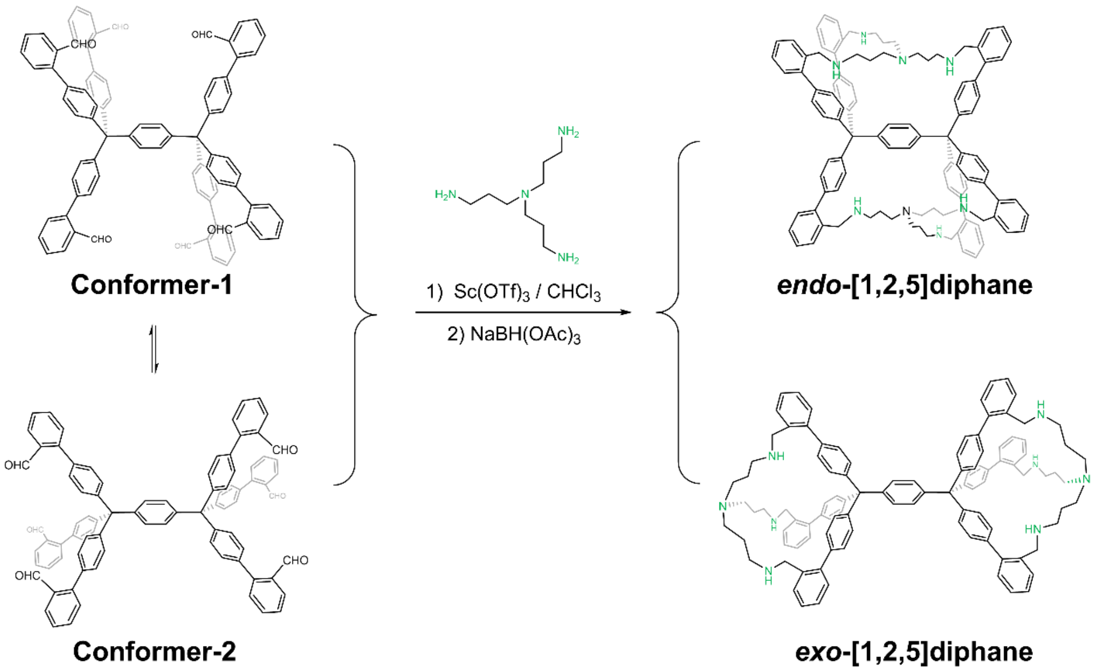

Supplementary Fig. 5 Conformer-1 and -2 capturing with *endo*- and *exo*-[1,2,5]diphane formation.

***endo*- and *exo*-[1,2,5]Diphane:** Into a 1000 mL flask was charged with 0.005 mol/L model molecule **1** (1.0 g, 0.84 mmol, 1 equiv.) 170 mL in $CHCl_3$, then a 0.005 mol/L solution of tris(3-aminopropyl)amine (CC-2) (319 mg, 1.7 mmol, 2 equiv.) 500 mL in $CHCl_3$ was added dropwise followed by addition of $Sc(OTf)_3$ (248 mg, 0.5 mmol, 0.6 equiv.) directly. The reaction mixture was stirred at room temperature for 4 h. Then, the product was reduced by $NaBH(OAc)_3$ (2.6 g, 12.6 mmol, 15 equiv.) overnight, the excess reductant $NaBH(OAc)_3$ was filtered off under vacuum and the solution was quenched with NaOH solution (2 M, 200 mL), extracted with $CHCl_3$ (3×200 mL), dried over anhydrous Na_2SO_4, and concentrated to give the crude product. Purification by flash column chromatography (DCM/MeOH/NH_3(aq) = 50:2:3, v/v/v) afforded the *endo*-[1,2,5]diphane (720 mg, 59%) and *exo*-[1,2,5]diphane (200 mg, 16%) as white solids. Similarly, NMR spectroscopy of *endo*-[1,2,5]diphane was conducted with additional trifluoroacetic acid (TFA) for better solubility in MeOD.

The structural characterization of *endo*-[1,2,5]diphane is as follows: ^{1}H NMR (400 MHz, MeOD), δ = 7.64–7.32 (m, 52H), 4.42 (s, 4H), 4.37 (m, 12H), 3.02 (br, 8H), 2.85–2.81 (m, 8H), 2.65 (br, 8H), 1.97 (br, 8H), 1.76 (br, 4H). ^{13}C NMR (101 MHz,

$CDCl_3$), δ =162.25, 161.88, 161.51, 161.14, 147.28, 145.47, 144.37, 144.27, 139.99, 139.57, 132.84, 132.77, 132.51, 132.05, 131.98, 131.29, 131.00, 130.32, 130.23, 129.89, 129.75, 129.52, 129.42, 121.72, 118.83, 117.45, 115.95, 114.63, 113.07, 65.51, 44.37, 22.48, 19.56. FT-IR (KBr, cm^{-1}): ν = 3357.8 (br), 2920.9 (s), 2850.6 (s), 2381.3 (br), 1659.4 (m), 1633.0 (m), 1458.4 (m), 1377.4 (m), 1265.6 (w), 1193.1 (w), 1114.0 (br), 1005.7 (w), 826.5 (m), 759.7 (m), 729.4 (m), 578.8 (w). HR-MS (MALDI): $C_{104}H_{107}N_8^+$ $(M+H)^+$, Calculated: 1468.8647; Found: 1468.8496.

The structural characterization of *exo*-[1,2,5]diphane is as follows: ^{1}H NMR (400 MHz, MeOD), δ = 7.63–7.37 (m, 54H), 4.17 (s, 12H), 2.87–2.91 (t, J = 16.0, 8.0 Hz, 12H), 2.33–2.37 (t, J = 16.0, 8.0 Hz, 12H), 1.67 (br, 12H). ^{13}C NMR (101 MHz, MeOD), δ = 163.16, 162.81, 147.78, 144.89, 143.98, 139.56, 132.77, 132.01, 131.76, 131.48, 130.91, 130.67, 129.83, 65.47, 53.31, 46.04, 25.08. FT-IR (KBr, cm^{-1}): ν = 3356.6 (br), 2954.3 (s), 2922.5 (s), 2851.2 (s), 2322.0 (s), 1673.7 (br), 1460.9 (m), 1377.9 (m), 1201.4 (w), 1019.7 (w), 823.1 (w), 763.9 (w). HR-MS (MALDI): $C_{104}H_{107}N_8^+$ $(M+H)^+$, Calculated: 1468.8647; Found: 1468.8496.

4.3 HPLC analysis of the distribution of the diphanes.

As the diphanes had a poor solubility in common organic solvents, we performed the HPLC analysis using their fully protonic products with additional trifluoacetic acid. As seen in Supplementary Fig. 6, the crude reaction mixture of molecule **1** with small sized CC-1 only yielded on diphane species, which was confirmed by HPLC of the purified *endo*-[1,2,4]diphane; while the crude reaction mixture of molecule **1** with large sized CC-2 yielded both *endo*-[1,2,5]diphane and *exo*-[1,2,5]diphane, which were assigned with the purified products.

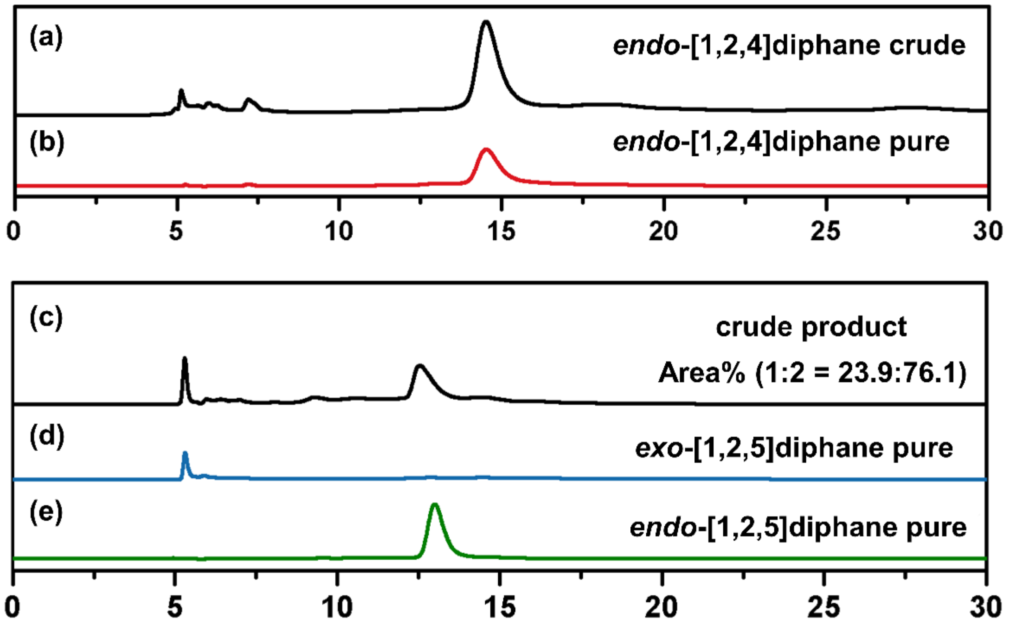

Supplementary Fig. 6 Stacked HPLC traces of (a) the crude reaction mixture of molecule **1** with small sized CC-1, (b) *endo*-[1,2,4]diphane, (c) the crude reaction mixture of molecule **1** with large sized CC-2, (d) *exo*-[1,2,5]diphane and (e) *endo*-[1,2,5]diphane.

5. Kinetic experiment and diphanes transformation

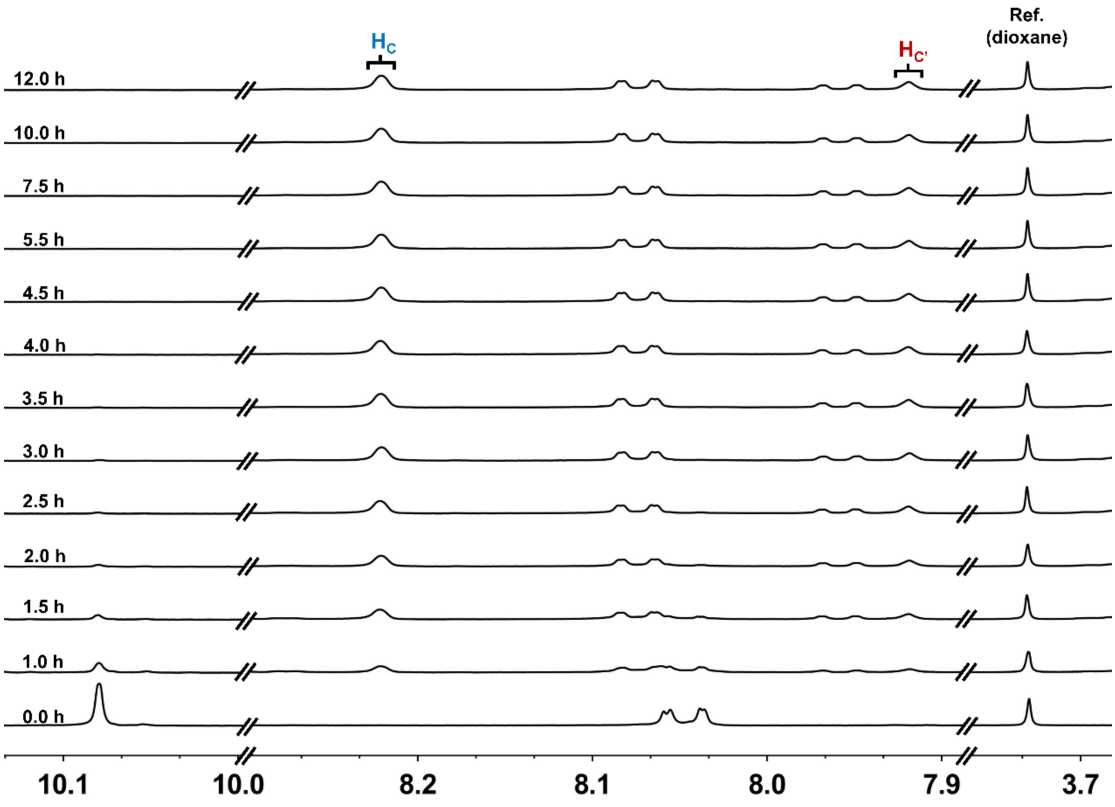

Supplementary Fig. 7 ^{1}H NMR study of the conformer capturing progress of Conformer-1 and -2 with large-sized CC-2 as a function of time.

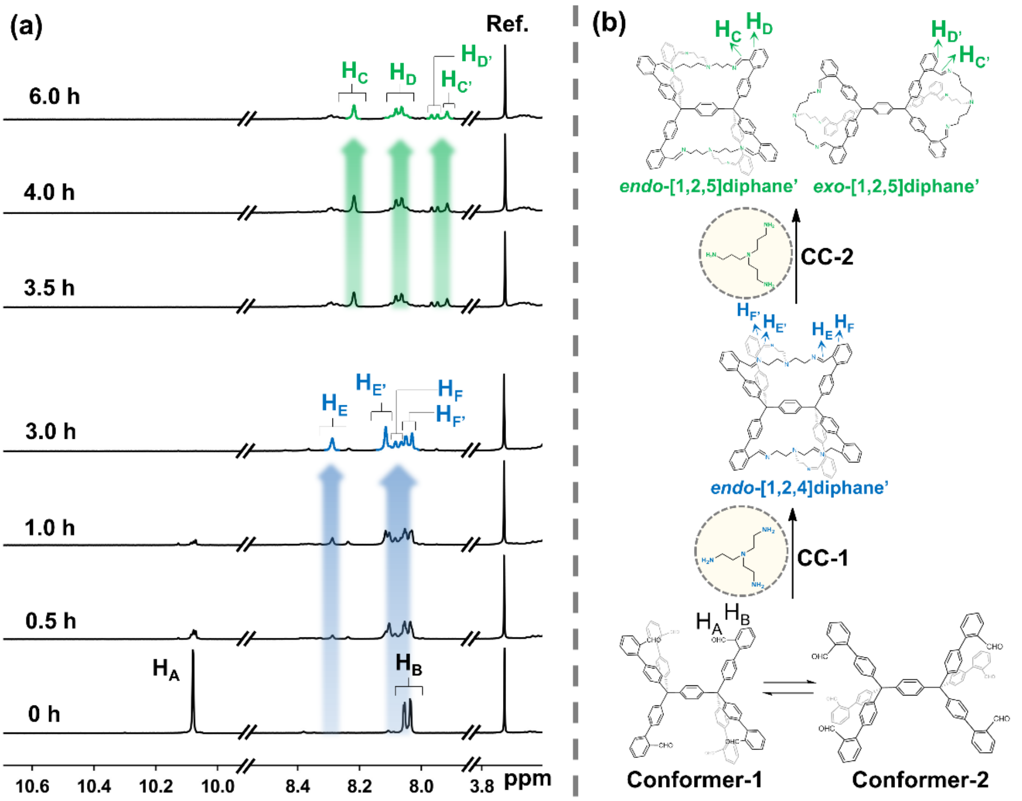

Supplementary Fig. 8 Transformation of *endo*-[1,2,4]diphane' to *endo*-[1,2,5]diphane' and *exo*-[1,2,5]diphane', followed by ^{1}H NMR study (400 Hz, $CDCl_3$, 25 ºC). (a) The ^{1}H NMR spectra suggest that Conformer-1 was captured exclusively with CC-1 (0-3.5 h), and the resulting *endo*-[1,2,4]diphane' was then transformed to *endo*-[1,2,5]diphane' and *exo*-[1,2,5]diphane' rapidly and completely by subsequent addition of CC-2 (3.5-6 h). (b) The reaction and chemical structures with the corresponding protons are given for reference.

A parellel study was also conducted to compare the thermodynamic stability of *endo*-[1,2,4]diphane' formed with small-sized CC-1, and *endo*-[1,2,5]diphane' and *exo*-[1,2,5]diphane' formed with large-sized CC-2 (Supplementary Fig. 8). CC-1 was first added to the solution of molecule **1**, forming exclusively *endo*-[1,2,4]diphane', as revealed by ^{1}H NMR (0-3 h). Large-sized CC-2 was then introduced to this reaction mixture. Within an additional 0.5 h, the peaks of *endo*-[1,2,4]diphane' completely disappeared in the ^{1}H NMR spectrum (3.5 h), while the formation of *endo*-[1,2,5]diphane' and *exo*-[1,2,5]diphane' was observed. No inverconversion between these three diphanes was noticed with prolonged reaction time (3.5-6 h). This result clearly indicates that *endo*-[1,2,5]diphane' and *exo*-[1,2,5]diphane' are thermodynamically more stable than *endo*-[1,2,4]diphane'.

6. Theoretical calculations

6.1 DFT calculations

All of the calculations reported here were performed with the Gaussian09 program.[7] Geometry optimization of all structures was carried out at B3LYP/6-31G* theoretical level including Grimme's dispersion correction.[8-13] After the geometry optimization was performed, analytical vibration frequencies were calculated at the same level to determine the nature of the located stationary point. In the meanwhile, the PCM solvation model was used to deal with the solvation effect. In the PCM method, the solvent was treated as an infinite continuum dielectric and the solvent response to the presence of the electric field generated by the solute charge distribution (assumed to be contained in a volume of known form and dimension, the molecular cavity) was represented in terms of an apparent charge spread on the cavity surface.

Here, all the calculations were carried out in chloroform phase unless otherwise specified. The results reveal that the energy difference between conformer-1 and conformer-2 is quite small (0.12 kJ/mol, Table 1). According to the Grimme's work,[14] B3LYP-D3 is indeed not the overall applicable functional, another M06-2X-D3 hybrid functionals [15] was therefore employed to check whether this energy difference between the two conformers is valid. We found that the energy difference of the Conformer-1 and -2 calculated with B3LYP-D3 (0.12 kJ/mol) and M062X-D3 (-0.72 kJ/mol) functionals is very similar within the tolerant range of experimental error (Table 1), which therefore confirms their fast interchange in $CHCl_3$. According to Grimme *et al.*, among all tested 23 hybrids M062X-D3 is statistically the best of all hybrids. However, they also observed SCF-convergence problems for all Minnesota functionals, even for simple atomic systems. In our calculations, we also found that some of the optimized relaxed structures through M062X-D3 functional were indeed difficult to converge. Thus, the results reported in this paper were all calculated by B3LYP-D3 method in chloroform unless with specific instructions.

Supplementary Table 1 The calculated conformation energy with B3LYP and M062X functionals.

	B3LYP/6-31G* (Gas)	B3LYP-D3/6-31G* ($CHCl_3$)	B3LYP-D3/6-31G* ($CHCl_3$, Free energy)	M062X-D3/6-31G* ($CHCl_3$)	M062X-D3/6-31G* ($CHCl_3$, Free energy)
Conformer-1 (kJ/mol)	0.00	0.00	0.00	0.00	0.00
Conformer-2 (kJ/mol)	-1.13	0.40	-0.12	0.45	0.72

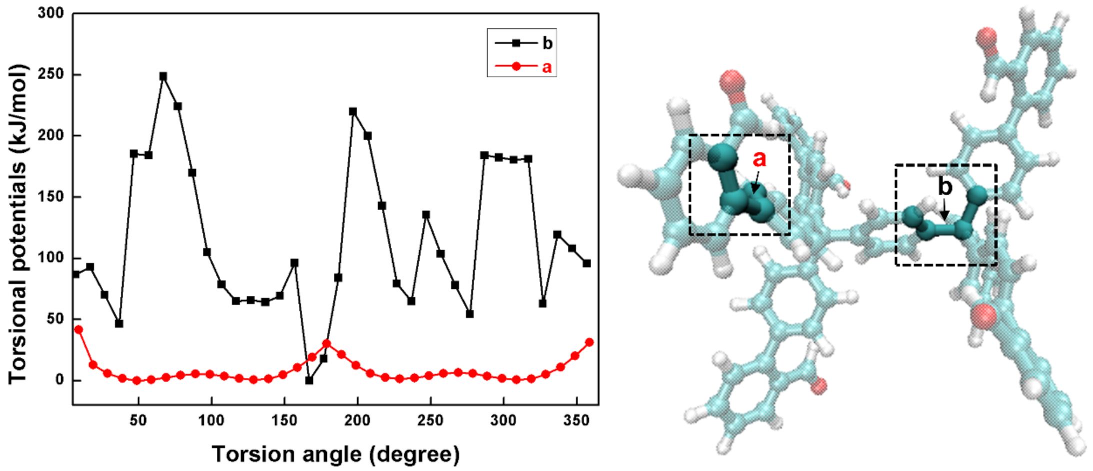

Supplementary Fig. 9 Variation of conformation energy with 1) the rotation around the inter-ring carbon-carbon bond a of the two aryl rings within a peripheral arm and 2) the rotation of the carbon-carbon bond b of the central benzene ring and the subsequent sp^3 carbon atom. The conformation energies were determined by relaxed potential energy scan.

We also calculated the torsional potentials of the rotation of C-C bonds a and b with relaxed potential energy scan. a represents the C-C bond of the two aryl rings within a peripheral arm and b indicates the C-C bond of the central benzene and the adjacent sp^3 carbon atom (Supplementary Fig. 9, right). The torsional potentials C-C bonds b fluctuate dramatically as a function of torsion angle, with the torsional potential up to 250 kJ/mol; while the torsional potential of C-C bonds a is considerably lower with a maximum value of *ca.* 30 kJ/mol (Supplementary Fig. 9, left). These results therefore suggest the interconversion of Conformer-1 and -2 is mainly due to the rotation of more liable C-C bonds a.

6.2 Molecular dynamics simulations

To investigate the structure transition dynamics of molecule **1** in chloroform, its simulation model and RESP partial charge distribution were constructed by using Antechamber module of AmberTools[16-17] and converted to Gromacs (version 2019.6) [18-19] input format for GAFF force filed[S12] through ACPYPE toolkit.[20] Then one molecule **1** was inserted in a cubic box of 6.28 nm× 6.28 nm× 6.28 nm with 3228 chloroform molecules. The GAFF force filed parameters of chloroform were taken from William A. Goddard III's work.[21] The solvated structures were subsequently subjected to 5000 steps of steepest descent minimization of the potential energy. Subsequently, 20 ns MD simulations were performed in the constant volume–constant temperature (NVT) ensemble using a V-rescale thermostat[22] to equilibrate the system at 303 K. Finally, 100 ns long constant pressure–constant temperature (NPT) equilibrium dynamics were carried out using a Parrinello-Rahman barostat and a V-rescale thermostat (0.5 ps for pressure relaxation and 0.1 ps time constant for heat bath coupling). The last 50 ns equilibration trajectory was further used to assess the structural properties. A leap-frog integrator was used for integrating the equations of motion. A time step of 1 fs was employed without any constraints. The cutoff radius of 10 Å with the nearest image convention was used for the vdW interaction calculations. The electrostatic interactions were evaluated by the particle mesh Ewald (PME) method with a direct space cutoff distance of 10 Å. All calculations were performed at a pressure P = 1.0 atm and a temperature T = 303.0 K.

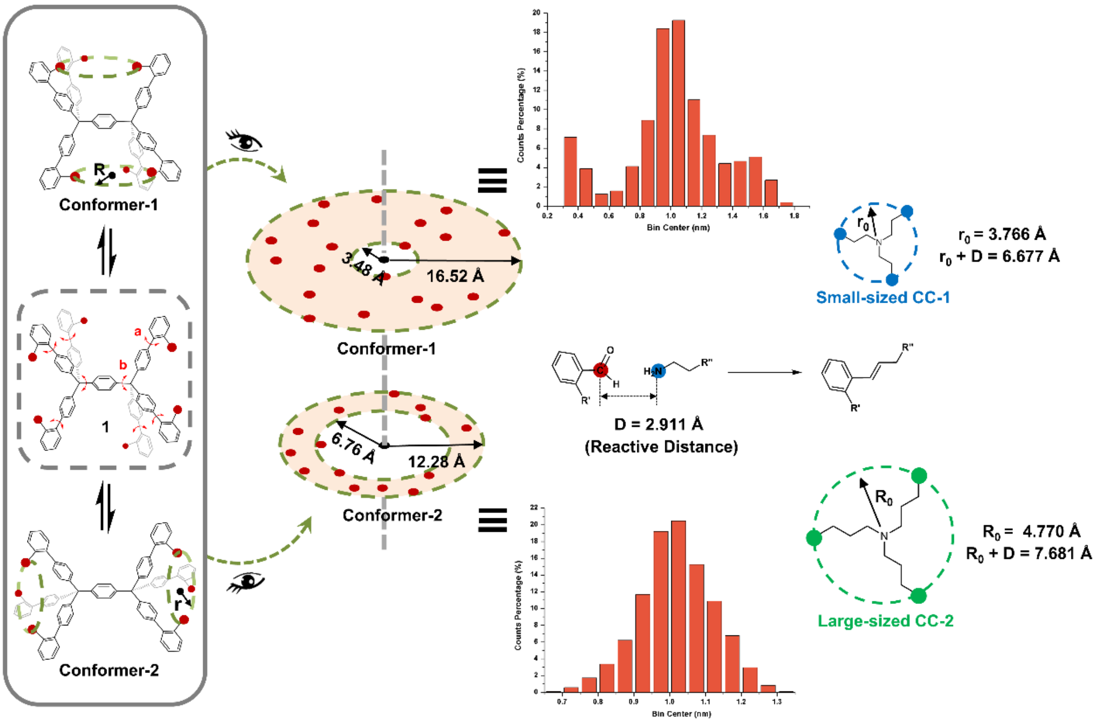

Supplementary Fig. 10 Model compound **1** exhibits infinite conformational isomers that fall into two types, namely Conformer-1 with a pair of upper and lower clefts and Conformer-2 with a pair of left and right clefts (left column). The region with the probability of containing formyl groups varies within the cleft of each conformer, determined by molecular dynamics simulations and illustrated with the projected circular rings that show the probability of formyl moieties (middle column). As the reactive distance of an amine and an aldehyde is esteemed to be 2.911 Å,[23] and the effective sizes of CC-1 and -2 were therefore calculated to be 6.677 and 7.681 Å, respectively (right column).

As the formyl groups are permanently moving, the region with the probability of containing formyl moieties form a circular ring within each cleft, and the range of the cleft of Conformer-1 and -2 is different. The region with formyl motion is similar to that of electron cloud surrounding an atomic nucleus, which spans from 3.48 to 16.52 Å for Conformer-1 and 6.76 to 12.28 Å for Conformer-2, respectively. We also calculated the sizes of CC-1 and -2, which are 3.766 and 4.770 Å, respectively. Taken into consideration of the reactive distance between an amine and an aldehyde (esteemed to be 2.911 Å[22]), the effective sizes of CC-1 and -2 were calculated to be 6.677 and 7.681 Å, respectively. It therefore means that both CC-1 and -2 can easily cover the "formyl cloud" of Conformer-1, producing both *endo*-[1,2,4]diphane and *endo*-[1,2,5]diphane. On the other hand, only large-sized CC-2 can theoretically reach all the formyl groups of Conformer-2, yielding the corresponding and *exo*-[1,2,5]diphane,

while these formyl moieties are out of reach for small-sized CC-1, unable to generate the corresponding *exo*-[1,2,4]diphane.

Strain Energy (SE) and Torsion Angles of diphanes calculations

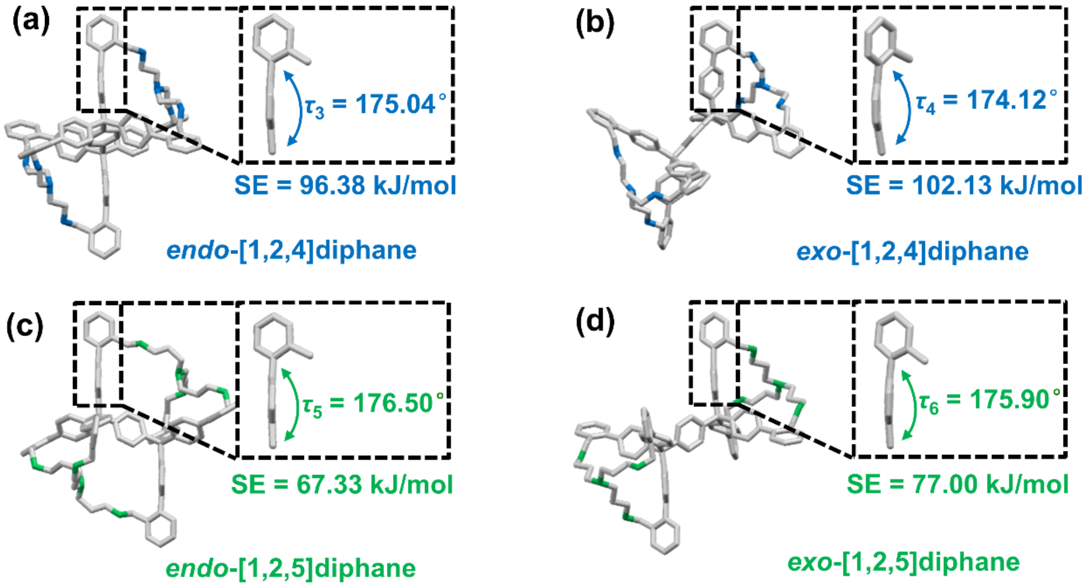

Diphane	Strain Energy (SE)	Torsion Angle (τ)	Relative Configuration Energy	Isolated Yield (%)
endo-[1,2,4]diphane	96.38 kJ/mol	175.04°	0.00 kJ/mol	64
exo-[1,2,4]diphane	102.13 kJ/mol	174.12°	51.02 kJ/mol	No
endo-[1,2,5]diphane	67.33 kJ/mol	176.50°	0.00 kJ/mol	59
exo-[1,2,5]diphane	77.00 kJ/mol	175.90°	23.86 kJ/mol	16

Supplementary Fig. 11 The strain energy (SE) and torsion angles of diphanes are shown in (a), (b), (c) and (d), which are corresponding to *endo*-[1,2,4]diphane, *exo*-[1,2,4]diphane, *endo*-[1,2,5]diphane and *exo*-[1,2,5]diphane, respectively (upper panel). The table (down panel) summaries SE, torsion angle, relative configuration energy and isolated yield of each diphane. The SE of *exo*-[1,2,4]diphane is the highest with 102.13 kJ mol^{-1}, larger than 96.38 kJ mol^{-1} for *endo*-[1,2,4]diphane, and *exo*-[1,2,4]diphane is significantly bent with the smallest torsion angle of τ_4 = 174.12º, smaller than 175.04º for *endo*-[1,2,4]diphane. When using the large-sized CC-2, the SE values of the resulting diphanes are considerably lower, with 67.33 kJ mol^{-1} for *endo*-[1,2,5]diphane, and 77.00 kJ mol^{-1} for *exo*-[1,2,5]diphane, respectively, echoed with their corresponding torsion angles. The calculated configuration energy show that *endo*-[1,2,4]diphane is 51.02 kJ/mol more stable than *exo*-[1,2,4]diphane, and *endo*-[1,2,5]diphane is 23.86 kJ/mol more stable than *exo*-[1,2,5]diphane, which are in line with the tendency of SE and torsion angle for diphanes. All the calculated results correlate well with the experimental yields obtained for the resulting diphanes.

The difference of thermodynamic stability of all diphanes were also studied by DFT calculation (B3LYP-D3 in PCM with chloroform as solvent), which is summarized in Supplementary Fig. 11. The strain energy (SE), torsion angle and relative configuration energy were computed to evaluate the possibility of forming a cage structure. SE is defined as the energy difference between the conformer in the cage and the optimized free conformer. It should be noted the end group of the conformers in the cage was reduced to aldehyde structures to maintain the equality of the calculations.

The SE of *exo*-[1,2,4]diphane is the highest with 102.13 kJ mol^{-1}, larger than 96.38 kJ mol^{-1} for *endo*-[1,2,4]diphane, and *exo*-[1,2,4]diphane is significantly bent with the smallest torsion angle of τ_4 = 174.12°, smaller than 175.04° for *endo*-[1,2,4]diphane. When using the large-sized CC-2, the SE values of the resulting *diphanes* are considerably lower, with 67.33 kJ mol^{-1} for *endo*-[1,2,5]diphane, and 77.00 kJ mol^{-1} for *exo*-[1,2,5]diphane, respectively, echoed with their corresponding torsion angles. The SE difference between *endo*-[1,2,4]diphane and *exo*-[1,2,4]diphane therefore presumably leads to the formation of the former as the major product. Considering no formation of *exo*-[1,2,4]diphane was observed while its SE is only slightly higher than that of *endo*-[1,2,4]diphane, we assume that a threshould effect might play a significant role that determines the formation of *diphanes* in the current study. The tendency of SE and torsion angle for *endo*-[1,2,4]diphane vs. *exo*-[1,2,4]diphane and *endo*-[1,2,5]diphane vs. *exo*-[1,2,5]diphane is also in line with that of their corresponding configuration energy, as the calculated results showed that *endo*-[1,2,4]diphane is 51.02 kJ/mol more stable than *exo*-[1,2,4]diphane, and *endo*-[1,2,5]diphane is 23.86 kJ/mol more stable than *exo*-[1,2,5]diphane. All the calculated results correlate well with the experimental yields obtained for the resulting diphanes.

7. X-ray crystallography

Single crystals of model molecule **1** suitable for X-ray crystallography were obtained by slow evaporation its $CHCl_3$ solution. CCDC deposition number 2033546.

Supplementary Table 2 Crystal data and structure refinement for molecule **1**

Identification code	Molecule **1**
Empirical formula	$C_{90}H_{62}Cl_{12}O_6$
Formula weight	1664.79
Temperature	170.0 K
Wavelength	1.34139 Å
Crystal system, space group	Triclinic, $P\bar{1}$
Unit cell dimensions	a = 12.344(2) Å, α = 94.414(6)°
	b = 13.319(2) Å, β = 109.530(5)°
	c = 15.047(3) Å, γ = 113.586(4)°
Volume	2072.3(6) $Å^3$
Z, Calculated density	1, 1.334 g/cm^3
Absorption coefficient	2.719 mm^{-1}
F (000)	854
Crystal size	0.08 × 0.06 × 0.06 mm^3
Theta range for data collection	2.795 to 55.046°
Limiting indices	-13 <= h <= 15, -16 <= k <= 16, -18 <= l <= 15
Reflections collected	23673
Independent reflections	7740 [R(int) = 0.0509]
Completeness to theta = 53.594°	98.2 %
Absorption correction	Semi-empirical from equivalents
Max and min. transmission	0.7508 and 0.6102
Refinement method	Full-matrix least-squares on F^2
Data / restraints / parameters	7740 / 0 / 487
Goodness-of-fit on F^2	1.000
Final R indices [I>2 sigma (I)]	R1 = 0.1366, wR2 = 0.3048
R indices (all data)	R1 = 0.1851, wR2 = 0.3388
Extinction coefficient	n/a
Largest diff. peak and hole	0.745 and -0.811 e. $Å^{-3}$

Single crystals of *endo*-[1,2,4]diphane suitable for X-ray crystallography were obtained by slow evaporation its THF solution with additional trifluoroacetic acid (TFA) for better solubility. CCDC deposition number 2033543.

Supplementary Table 3 Crystal data and structure refinement for *endo*-[1,2,4]diphane

Identification code	*endo*-[1,2,4]diphane
Empirical formula	$C_{118}H_{116}F_{18}N_8O_{14}$
Formula weight	2212.18
Temperature	173(2) K
Wavelength	1.54178 Å
Crystal system, space group	Triclinic, $P\bar{1}$
Unit cell dimensions	a = 13.713(2) Å, α = 74.959(18)°
	b = 15.458(3) Å, β = 69.23(2)°
	c = 16.447(5) Å, γ = 88.643(14)°
Volume	3139.1(13) $Å^3$
Z, Calculated density	1, 1.170 g/cm^3
Absorption coefficient	0.806 mm^{-1}
F (000)	1154
Crystal size	0.180 × 0.160 × 0.150 mm^3
Theta range for data collection	2.969 to 64.076°
Limiting indices	-15 <= h <= 15, -17 <= k <= 17, -19 <= l <= 19
Reflections collected	35362
Independent reflections	10326 [R(int) = 0.0960]
Completeness to theta = 64.076°	99.1 %
Absorption correction	Multi-scan
Max and min. transmission	0.7524 and 0.6163
Refinement method	Full-matrix least-squares on F^2
Data / restraints / parameters	10326 / 1 / 687
Goodness-of-fit on F^2	1.009
Final R indices [I>2 sigma (I)]	R1 = 0.0911, wR2 = 0.2611
R indices (all data)	R1 = 0.1357, wR2 = 0.3081
Extinction coefficient	n/a
Largest diff. peak and hole	0.529 and -0.477 e. $Å^{-3}$

Single crystals of *endo*-[1,2,5]diphane suitable for X-ray crystallography were obtained by slow evaporation its THF solution with additional trifluoroacetic acid (TFA) for better solubility. CCDC deposition number 2033544.

Supplementary Table 4 Crystal data and structure refinement for *endo*-[1,2,5]diphane

Identification code	*endo*-[1,2,5]diphane
Empirical formula	$C_{136}H_{146}F_{24}N_8O_{20}$
Formula weight	2668.60
Temperature	170.01 K
Wavelength	1.34139 Å
Crystal system, space group	Triclinic, $P\bar{1}$
Unit cell dimensions	a = 16.423(8) Å, α =77.61(2)°
	b = 16.785(11) Å, β = 66.924(14)°
	c = 19.465(9) Å, γ = 73.619(18)°
Volume	4703(4) $Å^3$
Z, Calculated density	1, 0.942 g/cm^3
Absorption coefficient	0.432 mm^{-1}
F (000)	1394
Crystal size	0.18 × 0.15 × 0.1 mm^3
Theta range for data collection	3.047° to 55.396°
Limiting indices	-19 <= h <= 20, -20 <= k <= 20, -23 <= l <= 23
Reflections collected	44705
Independent reflections	17594 [R(int) = 0.1319]
Completeness to theta = 53.594	98.6 %
Absorption correction	Semi-empirical from equivalents
Max and min. transmission	0.7508 and 0.2458
Refinement method	Full-matrix least-squares on F^2
Data / restraints / parameters	17594 / 0 / 847
Goodness-of-fit on F^2	0.974
Final R indices [I>2 sigma (I)]	R1 = 0.1470, wR2 = 0.3466
R indices (all data)	R1 = 0.2176, wR2 = 0.3928
Extinction coefficient	n/a
Largest diff. peak and hole	0.620 and -0.412 e. $Å^{-3}$

Single crystals of *exo*-[1,2,5]diphane suitable for X-ray crystallography were obtained by slow evaporation its THF solution with additional trifluoroacetic acid (TFA) for better solubility. CCDC deposition number 2033545.

Supplementary Table 5 Crystal data and structure refinement for *exo*-[1,2,5]diphane

Identification code	*exo*-[1,2,5]diphane
Empirical formula	$C_{184}H_{242}F_{24}N_8O_{32}$
Formula weight	3533.84
Temperature	173(2) K
Wavelength	1.54178 Å
Crystal system, space group	Triclinic, $P\bar{1}$
Unit cell dimensions	a = 16.2977(7) Å, α = 66.839(2)°
	b = 16.7904(8) Å, β = 74.146(2)°
	c = 19.3591(9) Å, γ = 82.849(3)°
Volume	4684.4(4) $Å^3$
Z, Calculated density	1, 1.253 g/cm^3
Absorption coefficient	0.849 mm^{-1}
F (000)	1874
Crystal size	0.200 × 0.200 × 0.200 mm^3
Theta range for data collection	2.561 to 65.534°
Limiting indices	-19 <= h <= 19, -19 <= k <= 19, -22 <= l <= 22
Reflections collected	78264
Independent reflections	15998 [R(int) = 0.0873]
Completeness to theta = 65.534°	99.0 %
Absorption correction	Semi-empirical from equivalents
Max and min. transmission	0.7526 and 0.5965
Refinement method	Full-matrix least-squares on F^2
Data / restraints / parameters	15998 / 264 / 1081
Goodness-of-fit on F^2	1.453
Final R indices [I>2 sigma (I)]	R1 = 0.1459, wR2 = 0.3884
R indices (all data)	R1 = 0.1864, wR2 = 0.4265
Extinction coefficient	n/a
Largest diff. peak and hole	0.790 and -0.444 e. $Å^{-3}$

Single crystals of Cage-2 suitable for X-ray crystallography were obtained by slow diffusion hexane into its solution in $CHCl_3$. CCDC deposition number 2033542.

Supplementary Table 6 Crystal data and structure refinement for Cage-2

Identification code	Cage-2
Empirical formula	$C_{58}H_{61}Cl_{4.50}N_4O$
Formula weight	989.63
Temperature	169.97 K
Wavelength	1.34139 Å
Crystal system, space group	Monoclinic, $P1\ 21/n\ 1$
Unit cell dimensions	a = 14.8065(3) Å, α = 90°
	b = 27.9439(5) Å, β = 100.2720(10)°
	c = 25.6301(5) Å, γ = 90°
Volume	10434.5(3) $Å^3$
Z, Calculated density	8, 1.260 g/cm^3
Absorption coefficient	1.745 mm^{-1}
F (000)	4172
Crystal size	0.1 × 0.03 × 0.02 mm^3
Theta range for data collection	2.802 to 55.010°
Limiting indices	-18 <= h <= 17, -34 <= k <= 29, -28 <= l <= 31
Reflections collected	79008
Independent reflections	19768 [R(int) = 0.1267]
Completeness to theta = 53.594°	99.9 %
Absorption correction	Semi-empirical from equivalents
Max and min. transmission	0.7508 and 0.4791
Refinement method	Full-matrix least-squares on F^2
Data / restraints / parameters	19768 / 0 / 1243
Goodness-of-fit on F^2	1.023
Final R indices [I>2 sigma (I)]	R1 = 0.0814, wR2 = 0.1930
R indices (all data)	R1 = 0.1610, wR2 = 0.2406
Extinction coefficient	n/a
Largest diff. peak and hole	0.944 and -0.796 $e.A^{-3}$

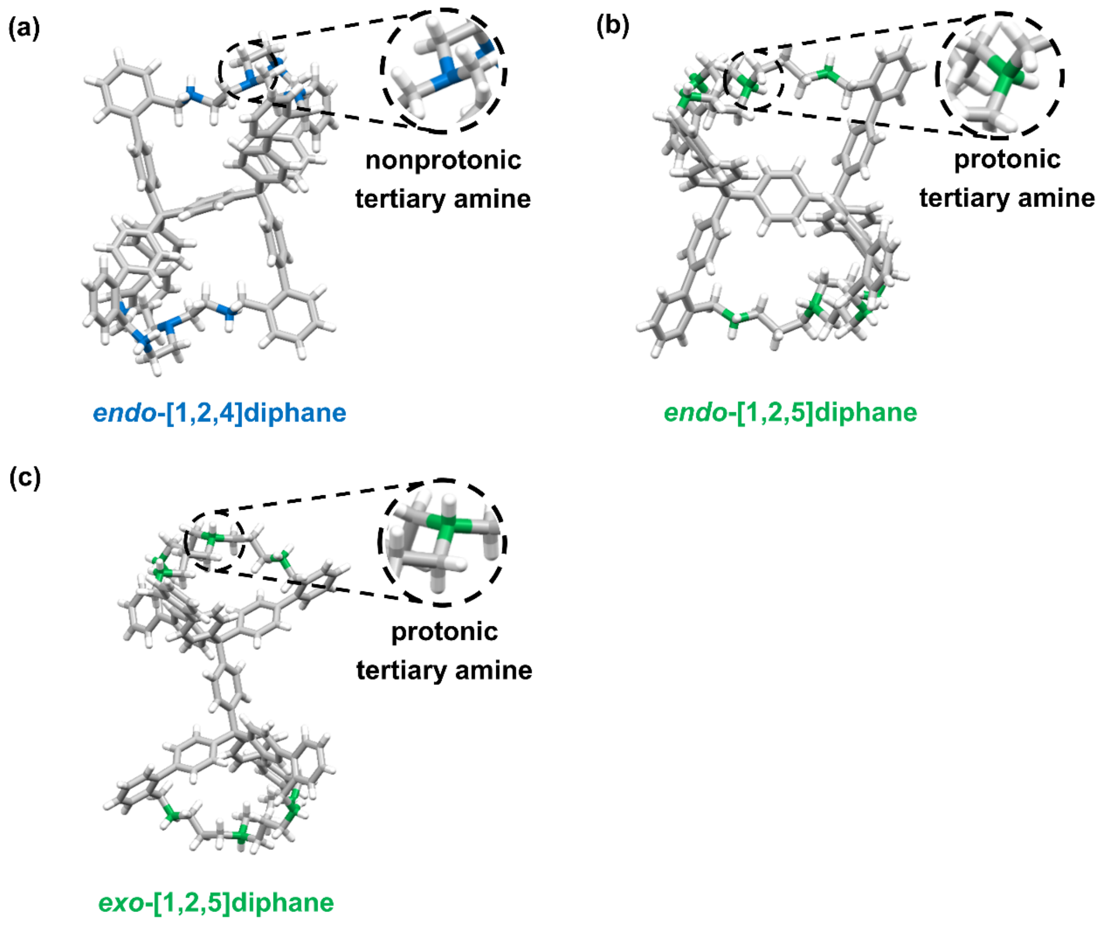

Supplementary Fig. 12 Single crystal structure of the diphanes, which are formed in THF with additional TFA. The zoom-in view shows the details of the tertiary amine area. (a) *endo*-[1,2,4]diphane, (b) *endo*-[1,2,5]diphane and (c) *exo*-[1,2,5]diphane. Solvent molecules are omitted and the protons are presented for clarity.

8. Powder X-ray diffraction pattern

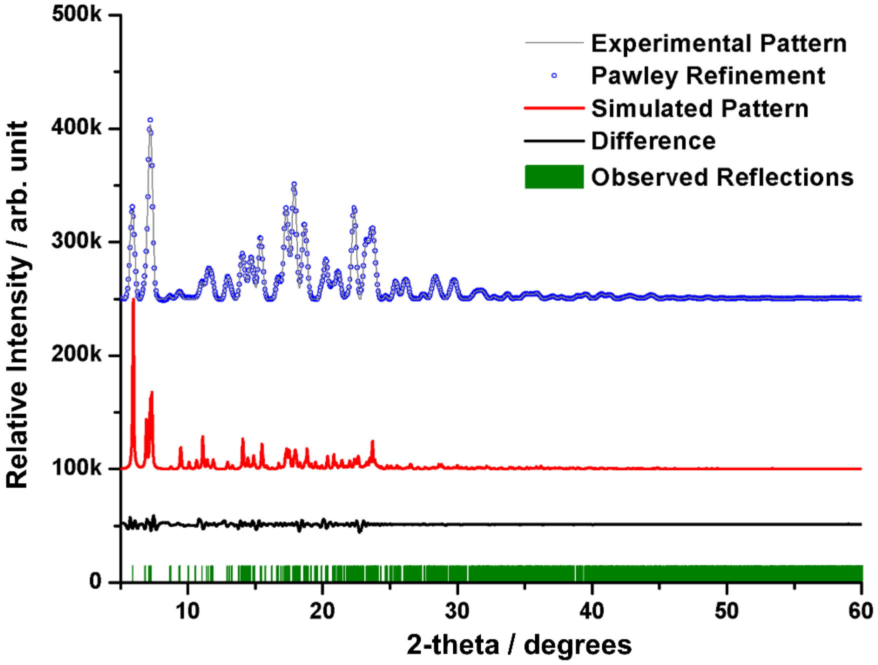

Supplementary Fig. 13 Powder X-ray pattern of *endo*-[1,2,4]diphane-TFA, experimental pattern (*grey line*), Pawley refinement (*blue circles*), simulated pattern (red line) and difference (*black line*). Profiles for Pawley refinement (R_{wp} = 3.56 %, R_p = 1.56 %) at 298 K (*a* = 13.717759, *b* = 15.453886, *c* = 16.448906 Å, *P*-1). The reflection positions are marked with green.2

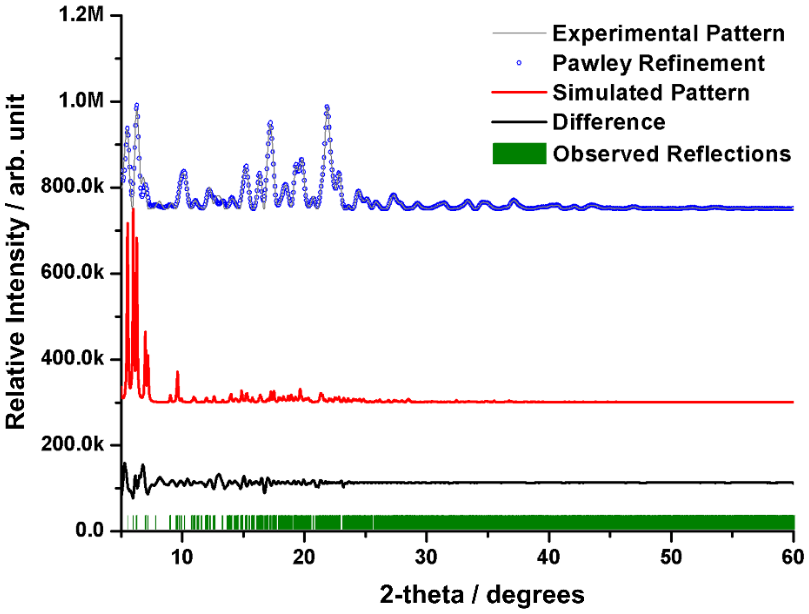

Supplementary Fig. 14 Powder X-ray pattern of *endo*-[1,2,5]diphane-TFA, experimental pattern (*grey line*), Pawley refinement (*blue circles*), simulated pattern (red line) and difference (*black line*). Profiles for Pawley refinement (R_{wp} = 3.31 %, R_p = 3.93 %) at 298 K (*a* = 16.421116, *b* = 16.786190, *c* = 19.463487 Å, *P*-1). The reflection positions are marked with green.

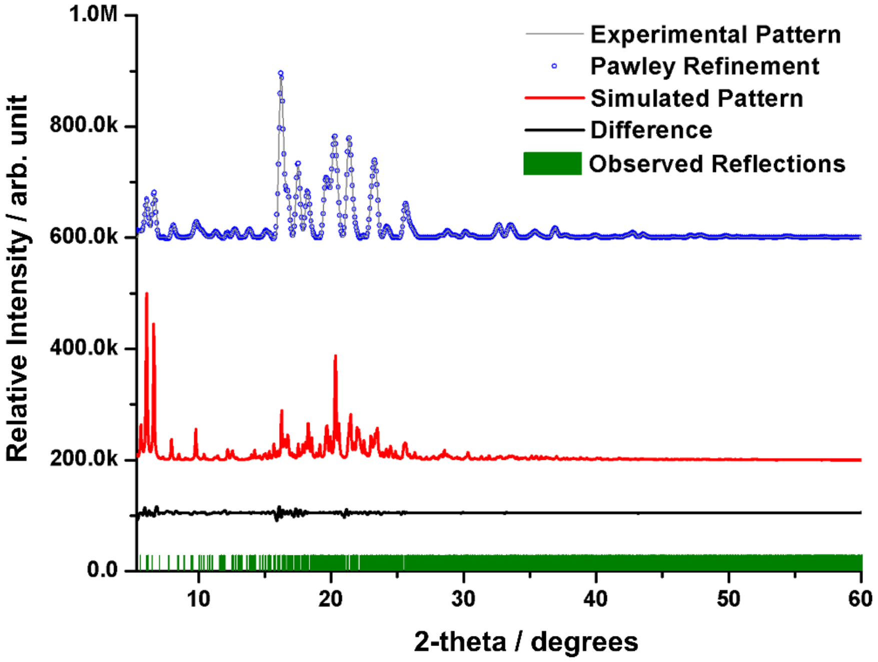

Supplementary Fig. 15 Powder X-ray pattern of *exo*-[1,2,5]diphane-TFA, experimental pattern (*grey line*), Pawley refinement (*blue circles*), simulated pattern (red line) and difference (*black line*). Profiles for Pawley refinement (R_{wp} = 3.36 %, R_p = 1.10 %) at 298 K (*a* = 16.303037, *b* = 16.787783, *c* = 19.356683 Å, *P*-1). The reflection positions are marked with green.

9. Proton conductivity

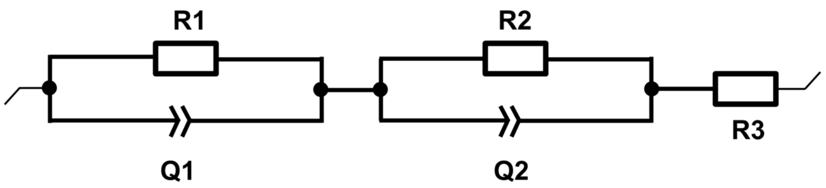

Supplementary Fig. 16. Equivalent circuit selection used to collect impedance data.

R1 is the contact resistance ascribed to grain boundary effects and happens at low frequencies, R2 is the resistance of sample, R3 is the contact resistance between sample pellet and electrode, Q1 and Q2 are constant phase element and the equivalent circuit selection is R1/Q1 + R2/Q2 + R3. The impedance calculation equation for a given frequency is as follows.

$$Z(f) = R3 + \frac{R2}{1 + j2\pi fR2Q2} + \frac{R1}{1 + j2\pi fR1Q1}$$

Supplementary Equation 1. The impedance calculation equation for a given frequency.

The ionic conductivity is calculated using the formula $\sigma = \frac{L}{RS}$ where σ is the ionic conductivity (S cm^{-1}), L is the electrodes distance (cm) and S is the cross sectional area of the sample pellet (cm^2).

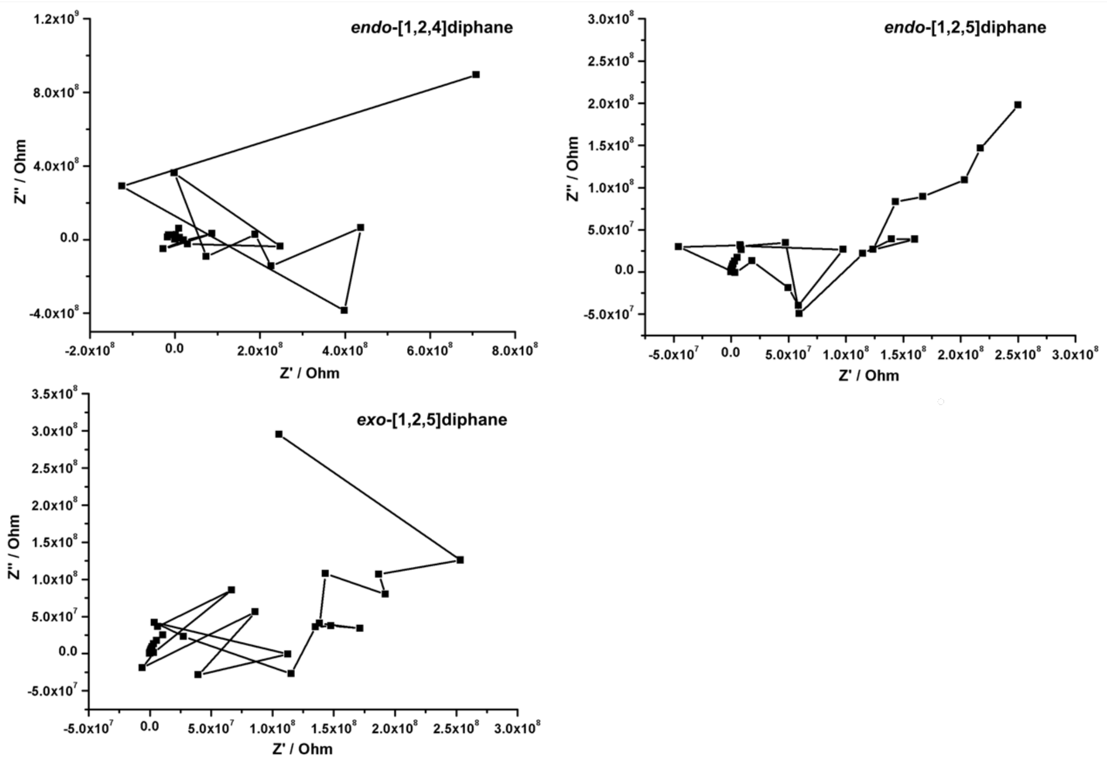

Supplementary Fig. 17 Nyquist plots of (a) *endo*-[1,2,4]diphane, (b) *endo*-[1,2,5]diphane and (c) *exo*-[1,2,5]diphane without doping of TFA.

Supplementary Table 7 Proton conductivities at different temperatures under the air humidity (48% RH).

	Temperature (℃)	30	40	50	60
***endo*-[1,2,4]diphane-TFA**	**R (Ω)**	2.01×10^{7}	1.67×10^{7}	1.43×10^{7}	1.23×10^{7}
	**σ (S cm^{-1})**	1.50×10^{-9}	1.80×10^{-9}	2.11×10^{-9}	2.44×10^{-9}
***endo*-[1,2,5]diphane-TFA**	**R (Ω)**	8.82×10^{3}	5.27×10^{3}	4.27×10^{3}	2.19×10^{3}
	**σ (S cm^{-1})**	3.17×10^{-6}	5.71×10^{-6}	7.04×10^{-6}	1.37×10^{-5}
***exo*-[1,2,5]diphane-TFA**	**R (Ω)**	3.00×10^{6}	2.40×10^{6}	2.19×10^{6}	1.75×10^{6}
	**σ (S cm^{-1})**	1.00×10^{-8}	1.25×10^{-8}	1.37×10^{-8}	1.72×10^{-8}

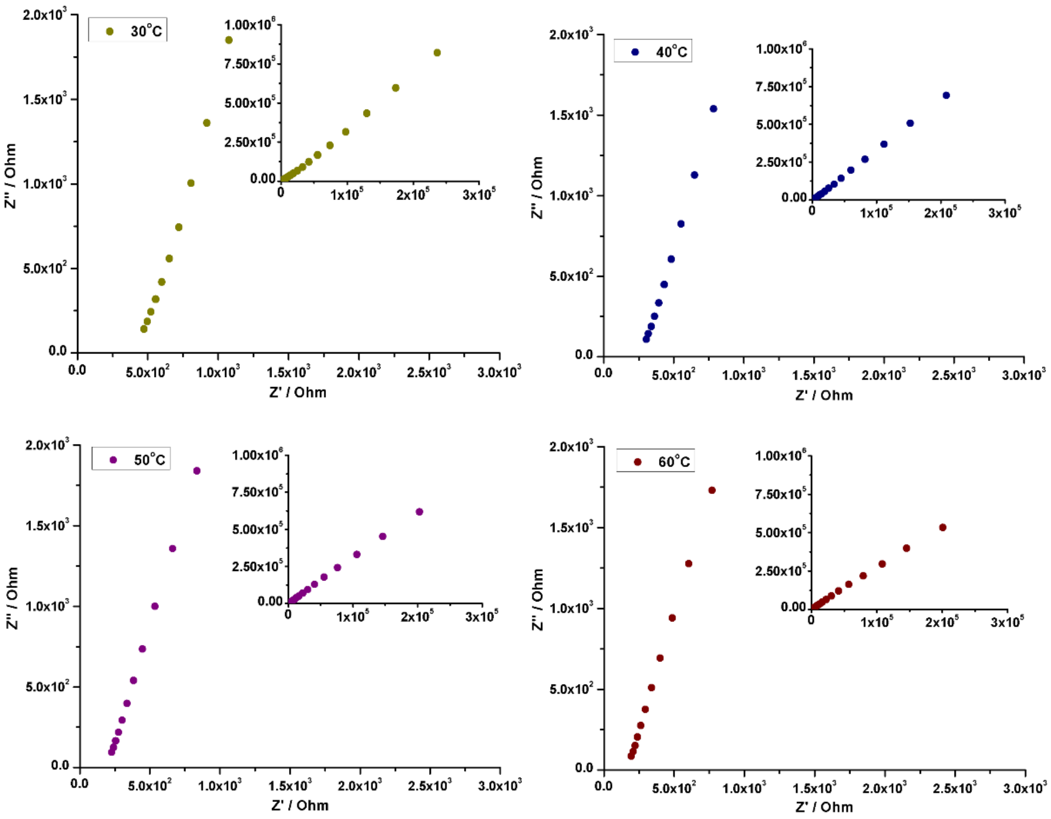

Supplementary Fig. 18 Nyquist plots of *endo*-[1,2,4]diphane-TFA at different temperatures under the air humidity (48% RH).

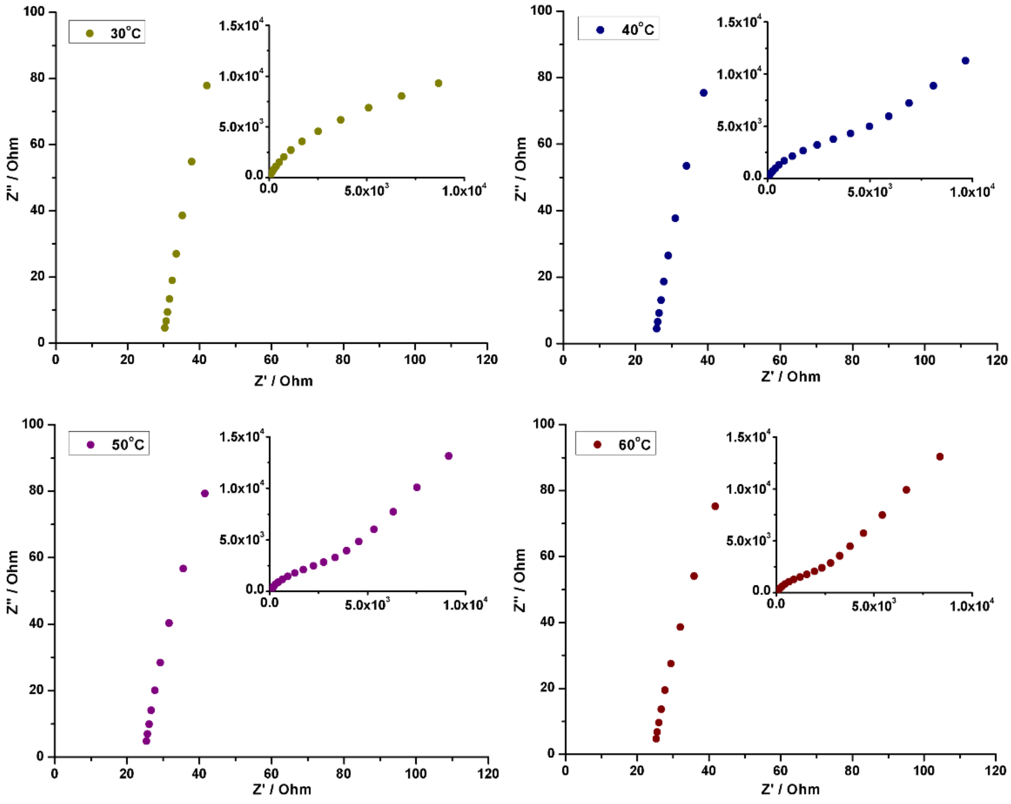

Supplementary Fig. 19 Nyquist plots of *endo*-[1,2,5]diphane-TFA at different temperatures under the air humidity (48% RH).

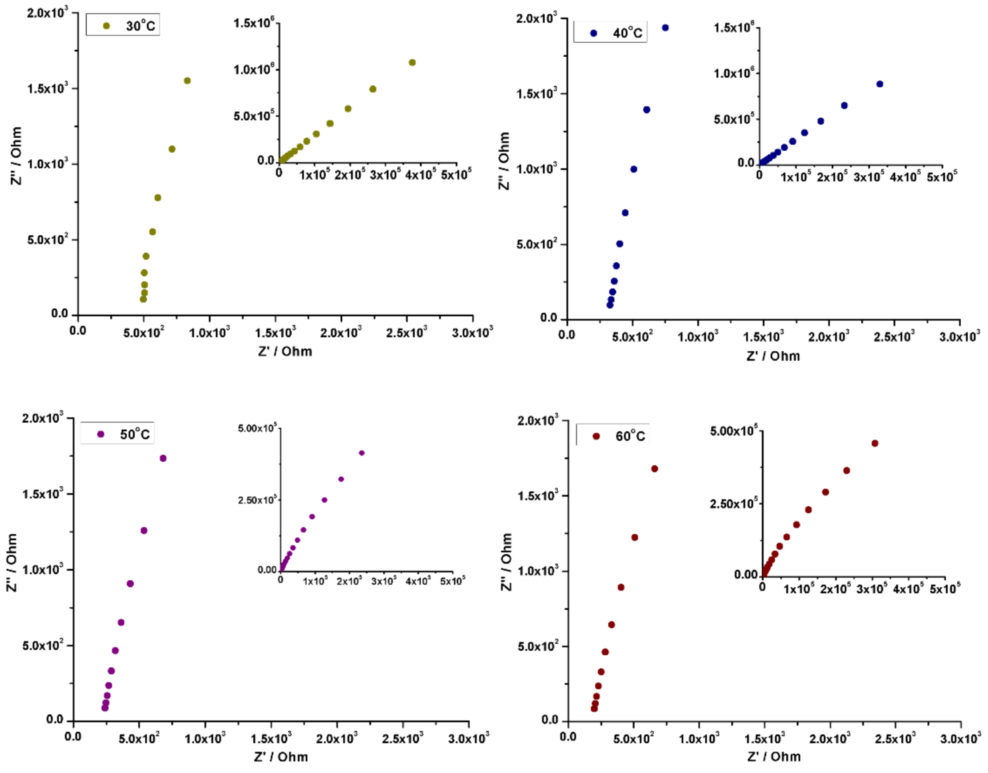

Supplementary Fig. 20 Nyquist plots of *exo*-[1,2,5]diphane-TFA at different temperatures under the air humidity (48% RH).

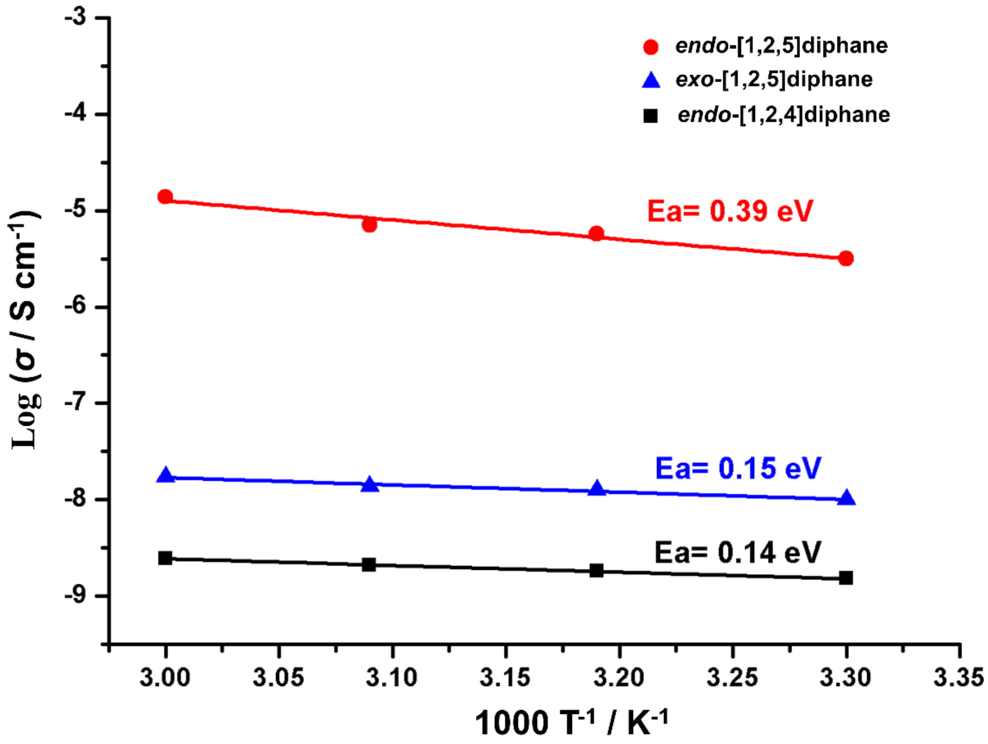

Supplementary Fig. 21 Arrhenius plots of diphanes-TFA and their corresponding activation energy. Temperature data were collected at the air humidity (48%) over the temperature range 303.15-333.15 K.

10. Solid-state NMR spectroscopy (ssNMR)

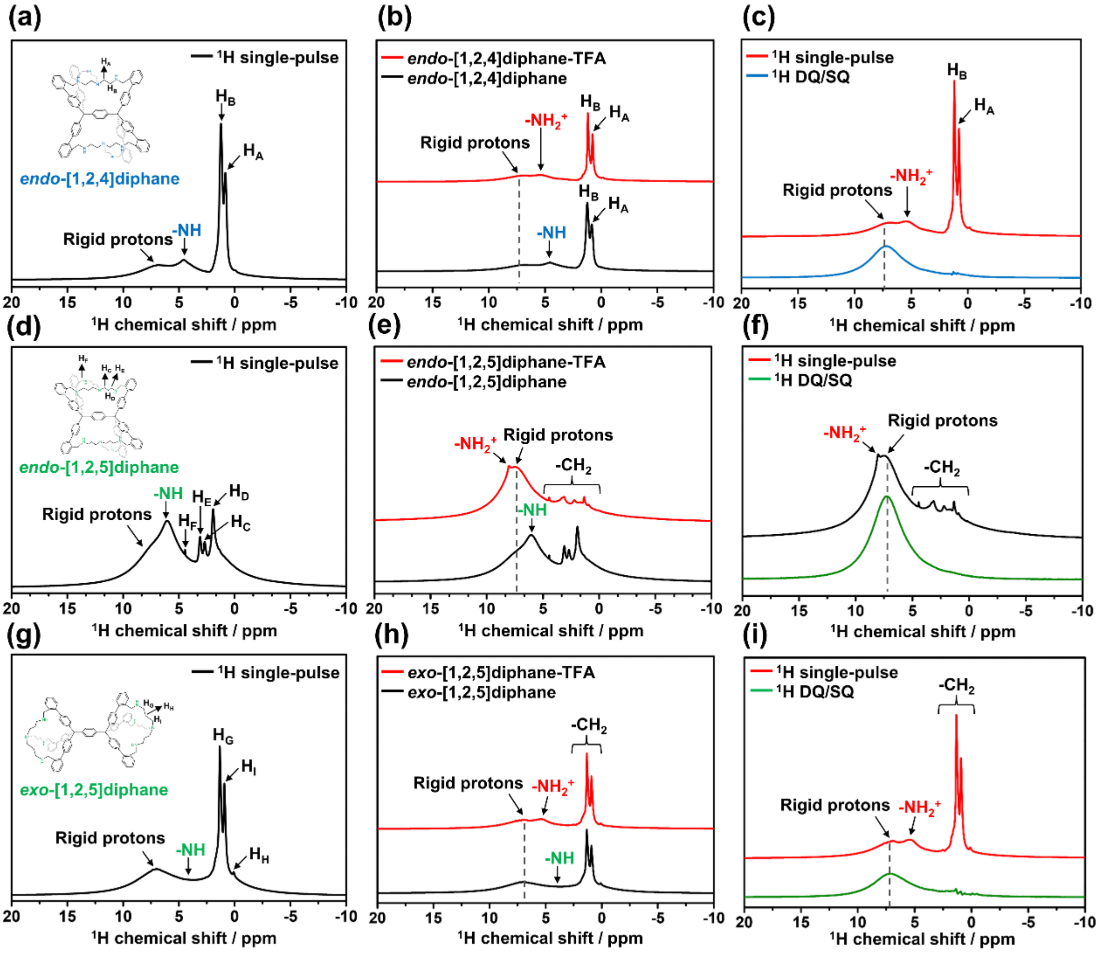

Supplementary Fig. 22 (a), (d) and (g) correspond to the 1D ^{1}H single-pulse spectrum of *endo*-[1,2,4]diphane, *endo*-[1,2,5]diphane and *exo*-[1,2,5]diphane. (b), (e) and (h) are the comparison of ^{1}H single-pulse spectra between diphane (black line) and diphane-TFA (red line). (c), (f) and (i) correspond to the comparison of ^{1}H single-pulse (red line) and ^{1}H DQ/SQ (blue and green) spectra for diphane-TFA. Diphane-TFA is protonated crystal powders of diphane, which are formed by slow evaporation of its THF solution with adding additional TFA.

11. Spectra

11.1 Mass spectra

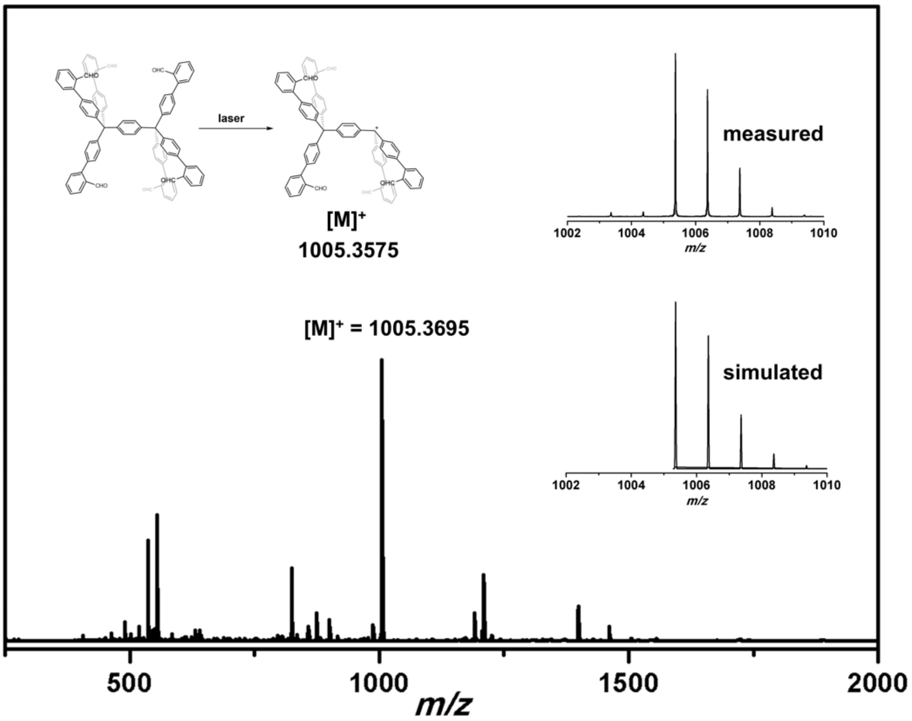

Supplementary Fig. 23 MALDI-TOF-MS of model molecule **1**. (positive-reflector mode, DCTB as matrix).

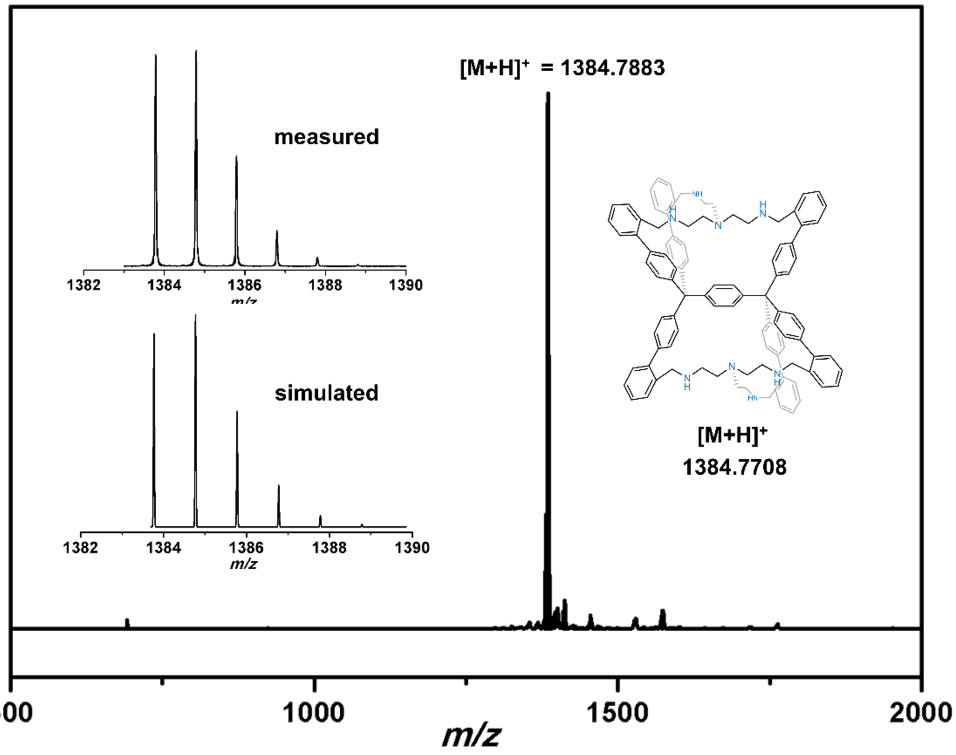

Supplementary Fig. 24 MALDI-TOF-MS of *endo*-[1,2,4]diphane. (positive-reflector mode, DCTB as matrix).

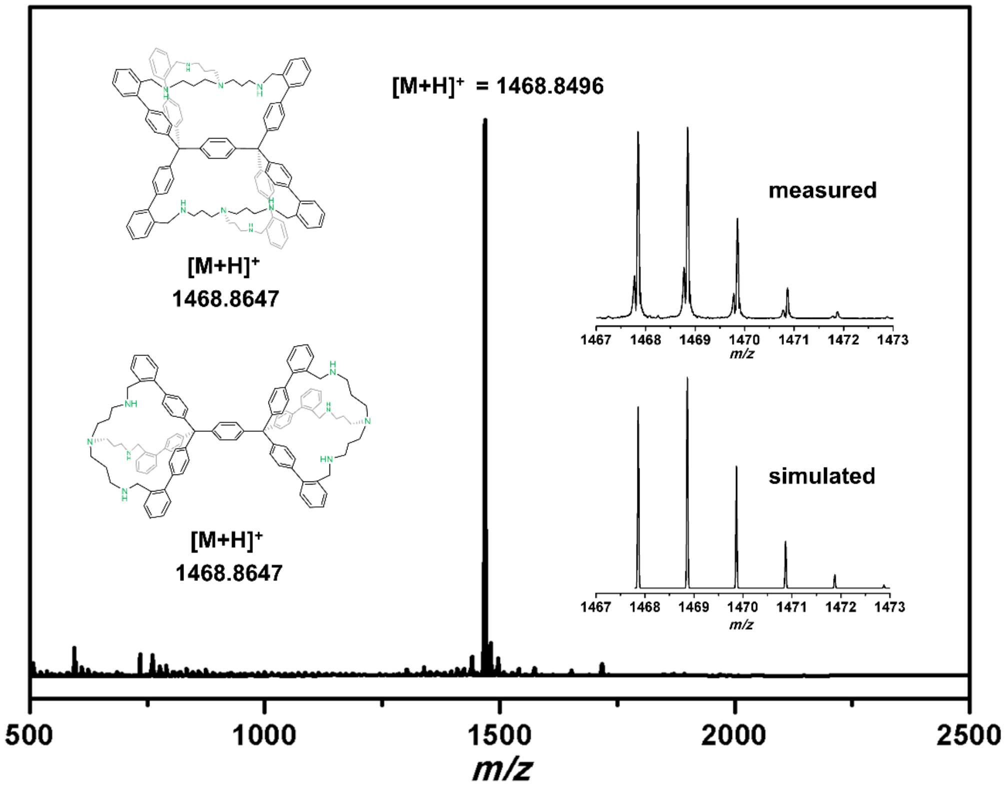

Supplementary Fig. 25 MALDI-TOF-MS of *endo*-[1,2,5]diphane and *exo*-[1,2,5]diphane. (positive-reflector mode, DCTB as matrix)

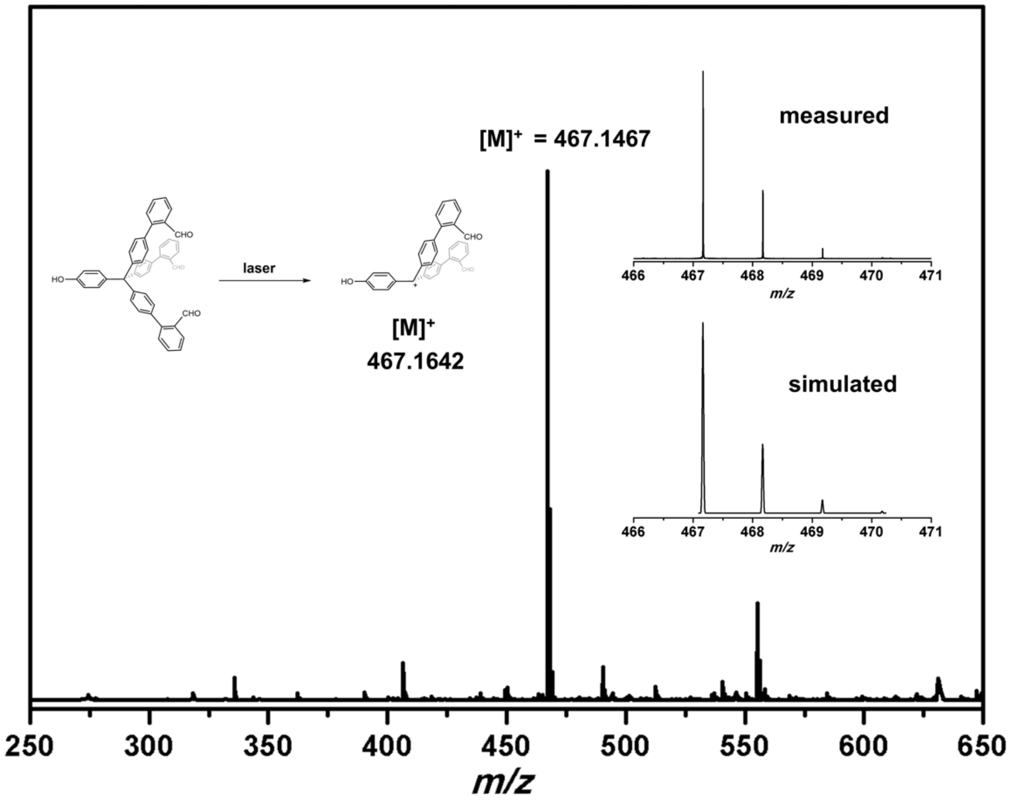

Supplementary Fig. 26 MALDI-TOF-MS of model molecule **2**. (positive-reflector mode, DCTB as matrix).

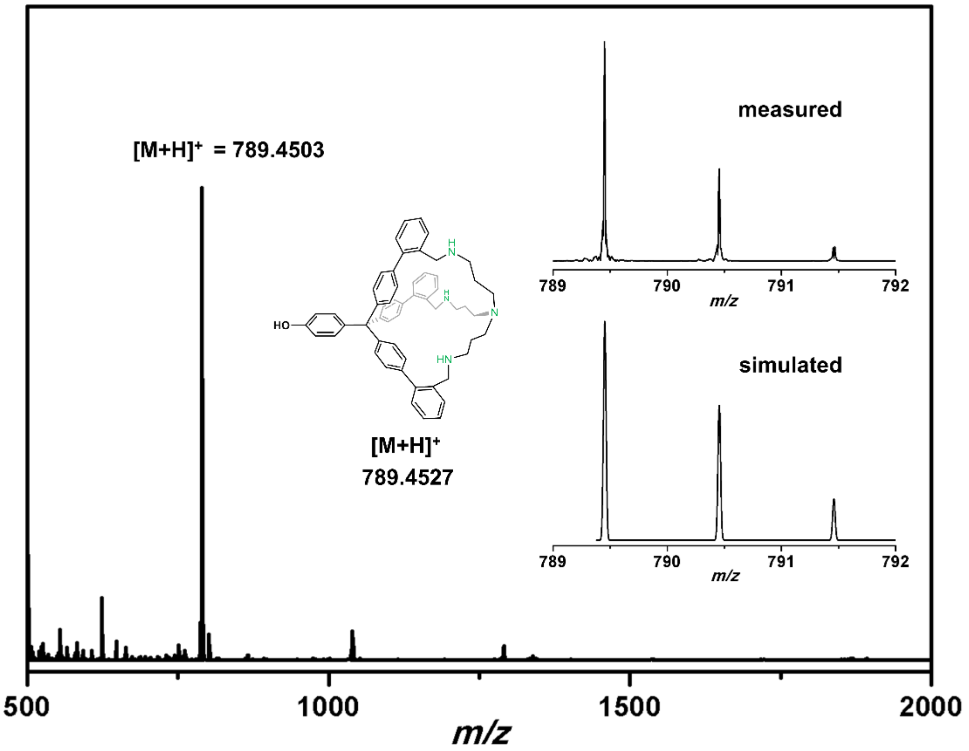

Supplementary Fig. 27 MALDI-TOF-MS of Cage-2. (positive-reflector mode, DCTB as matrix)

11.2 IR spectra

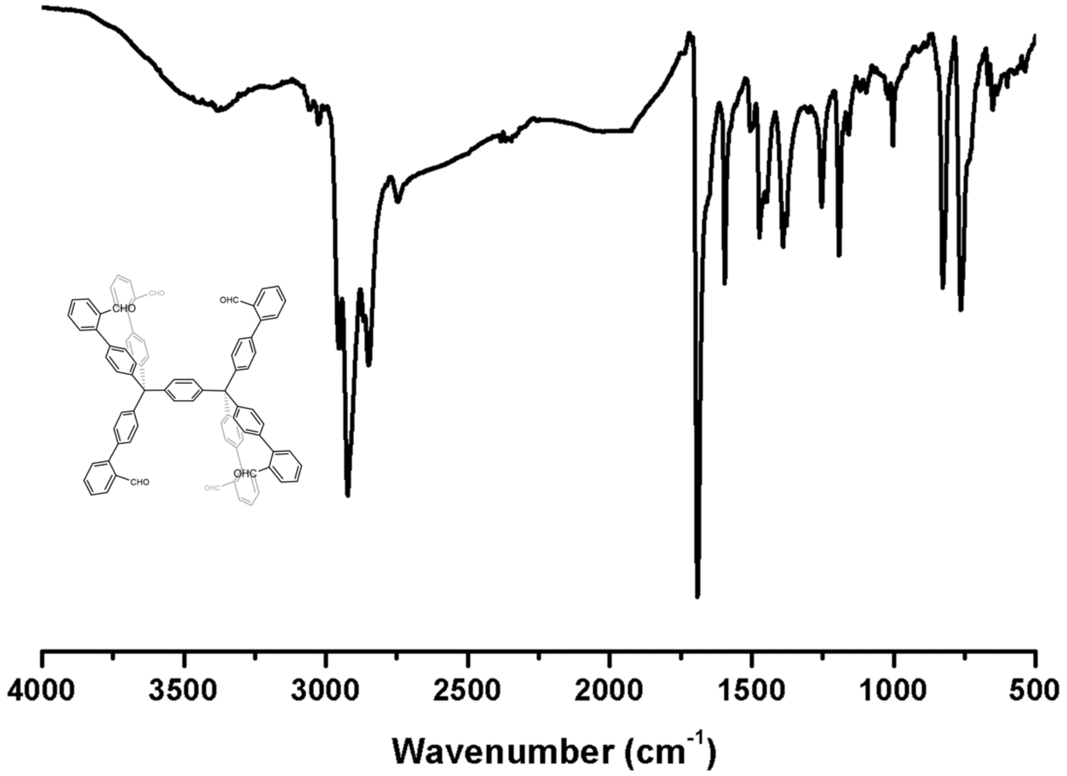

Supplementary Fig. 28 FTIR spectrum of model molecule **1**.

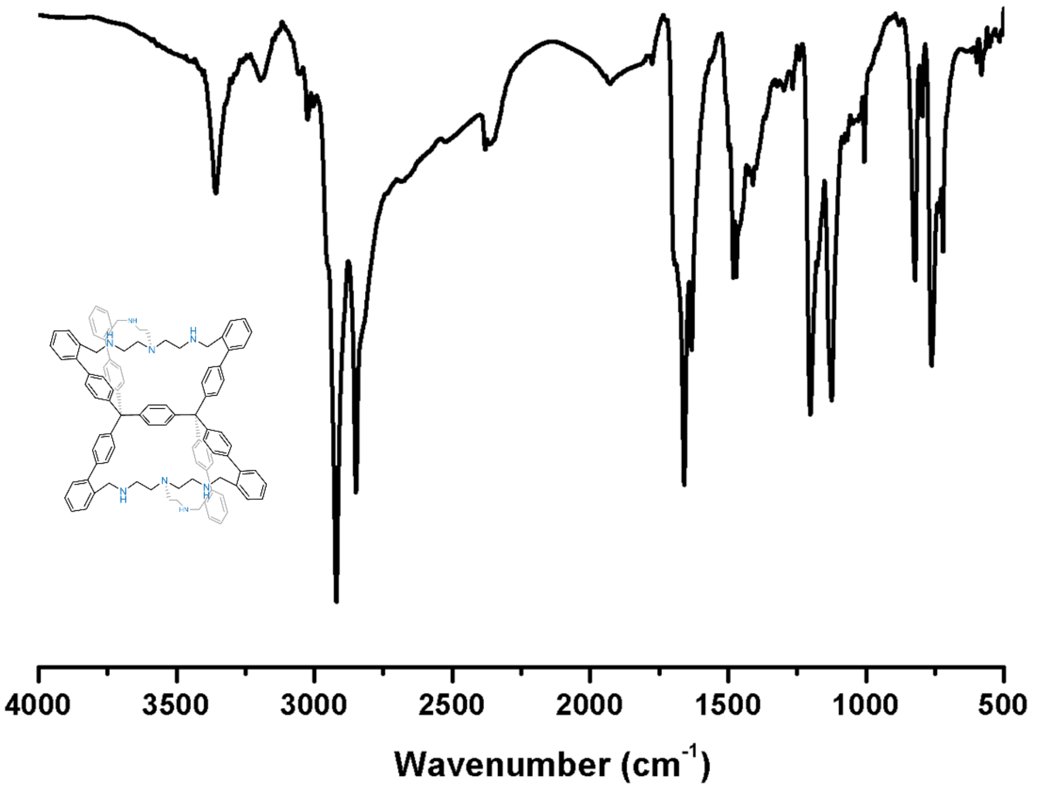

Supplementary Fig. 29 FTIR spectrum of *endo*-[1,2,4]diphane.

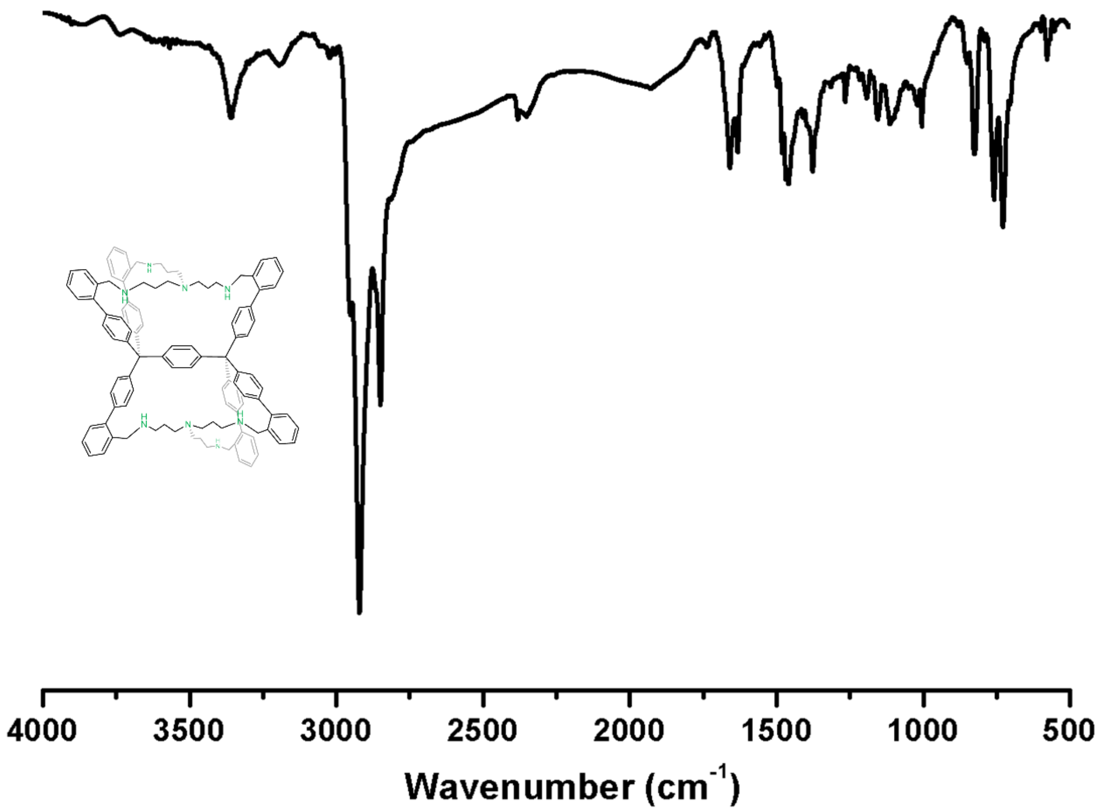

Supplementary Fig. 30 FTIR spectrum of *endo*-[1,2,5]diphane.

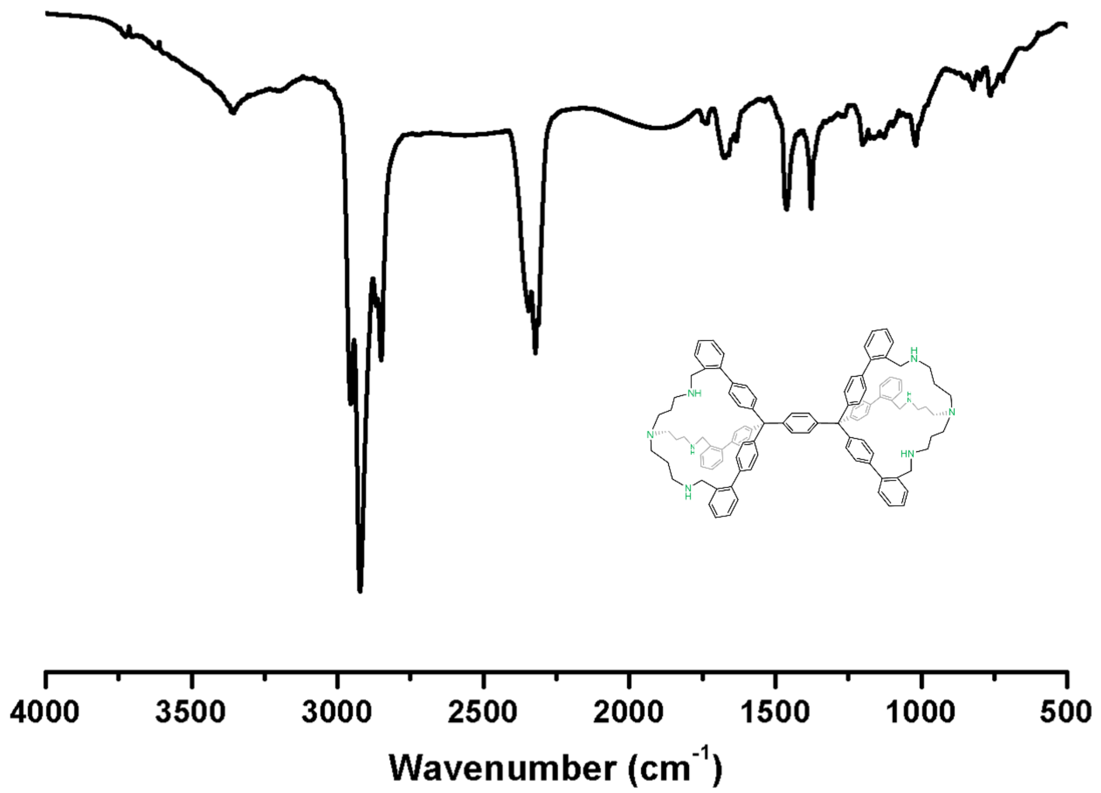

Supplementary Fig. 31 FTIR spectrum of *exo*-[1,2,5]diphane.

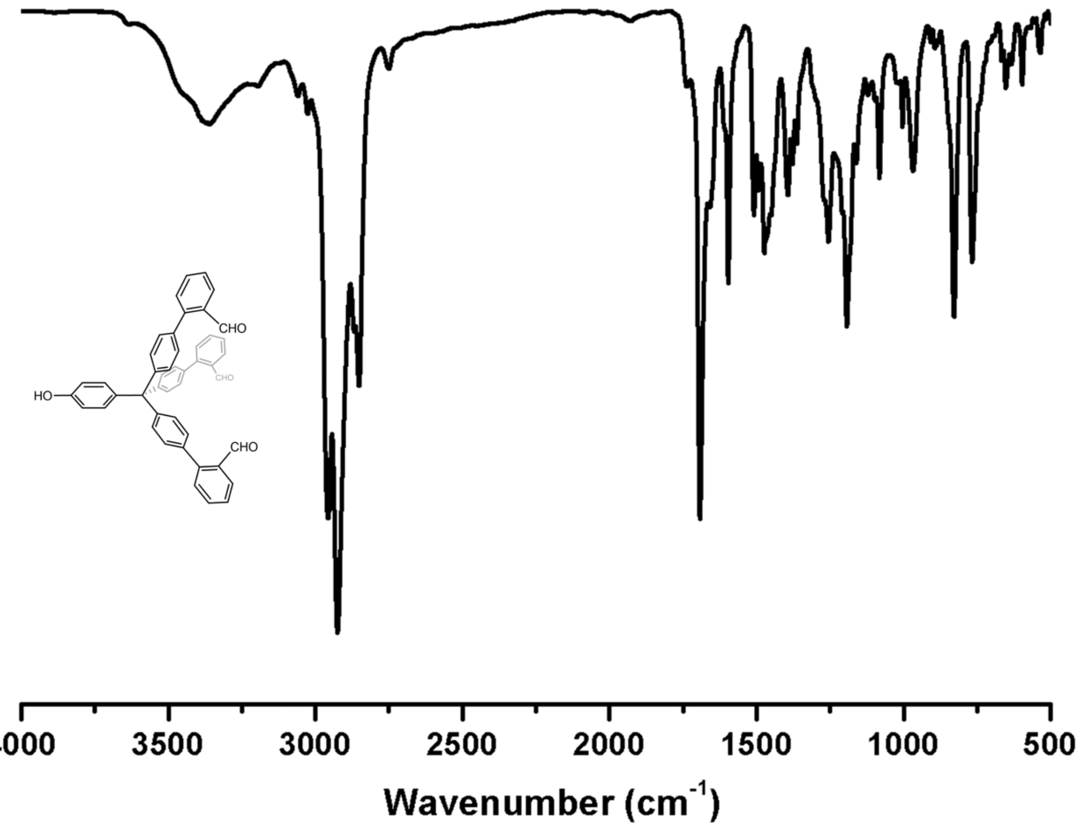

Supplementary Fig. 32 FTIR spectrum of model molecule **2**.

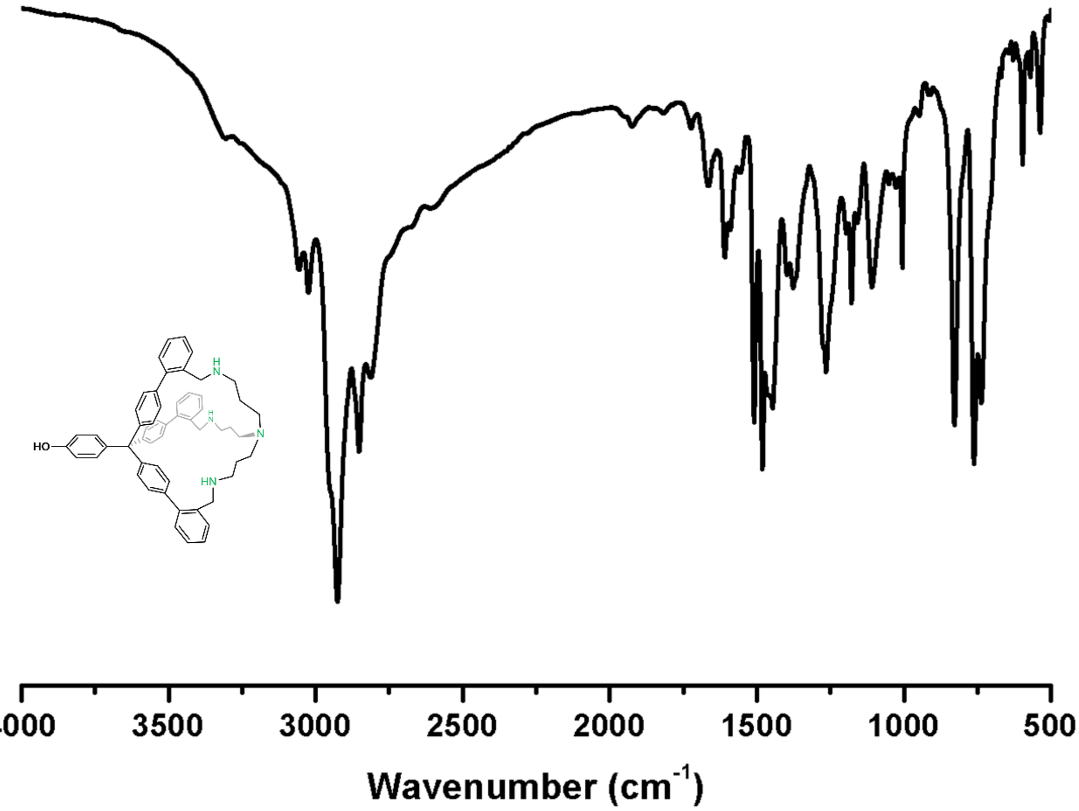

Supplementary Fig. 33 FTIR spectrum of model Cage-2.

11.3 NMR spectra

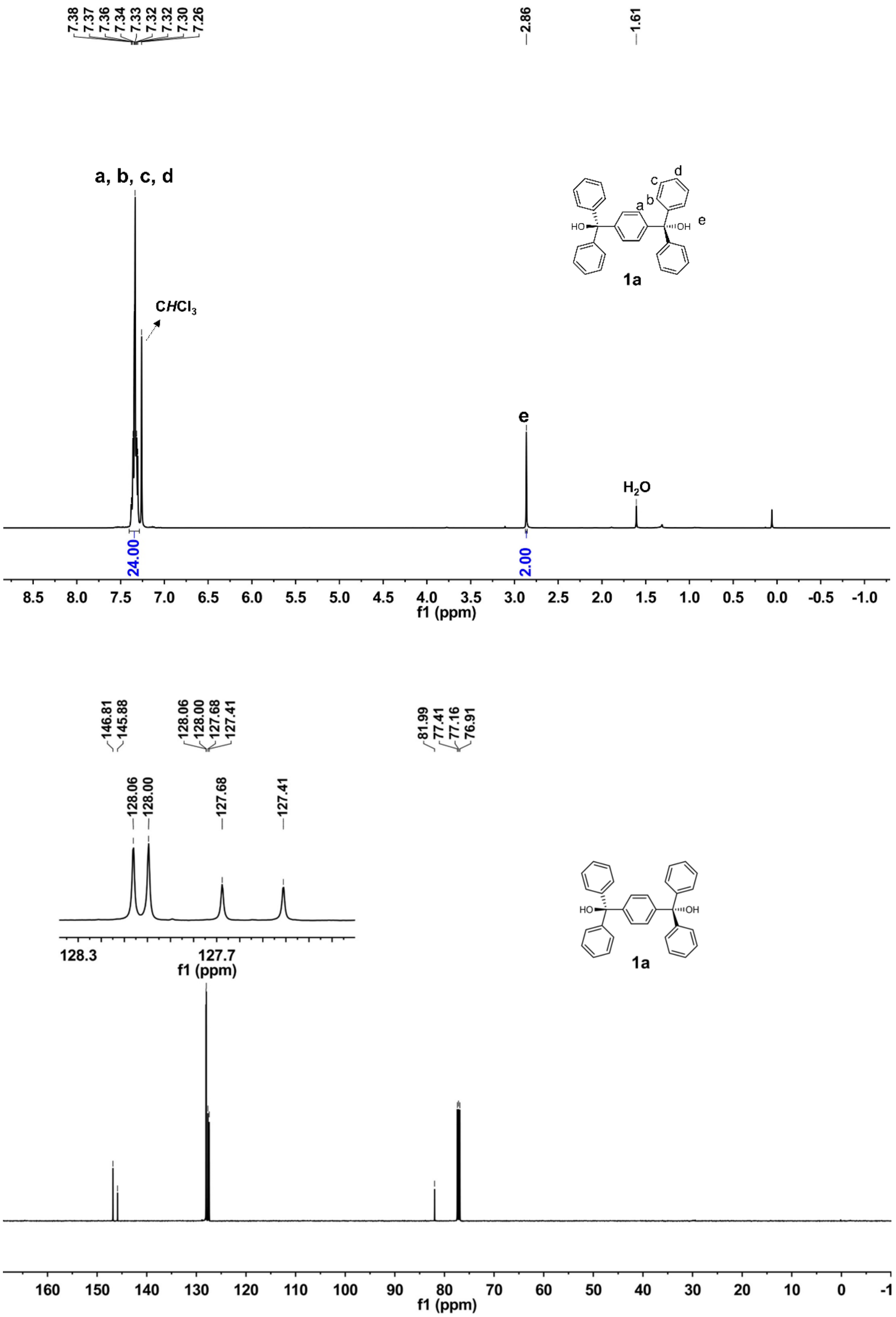

Supplementary Fig. 34 ^{1}H NMR (top, 400 MHz) and ^{13}C NMR (bottom, 101 MHz) spectra of 1,4-phenylenebis (diphenylmethanol) **1a** in $CDCl_3$.

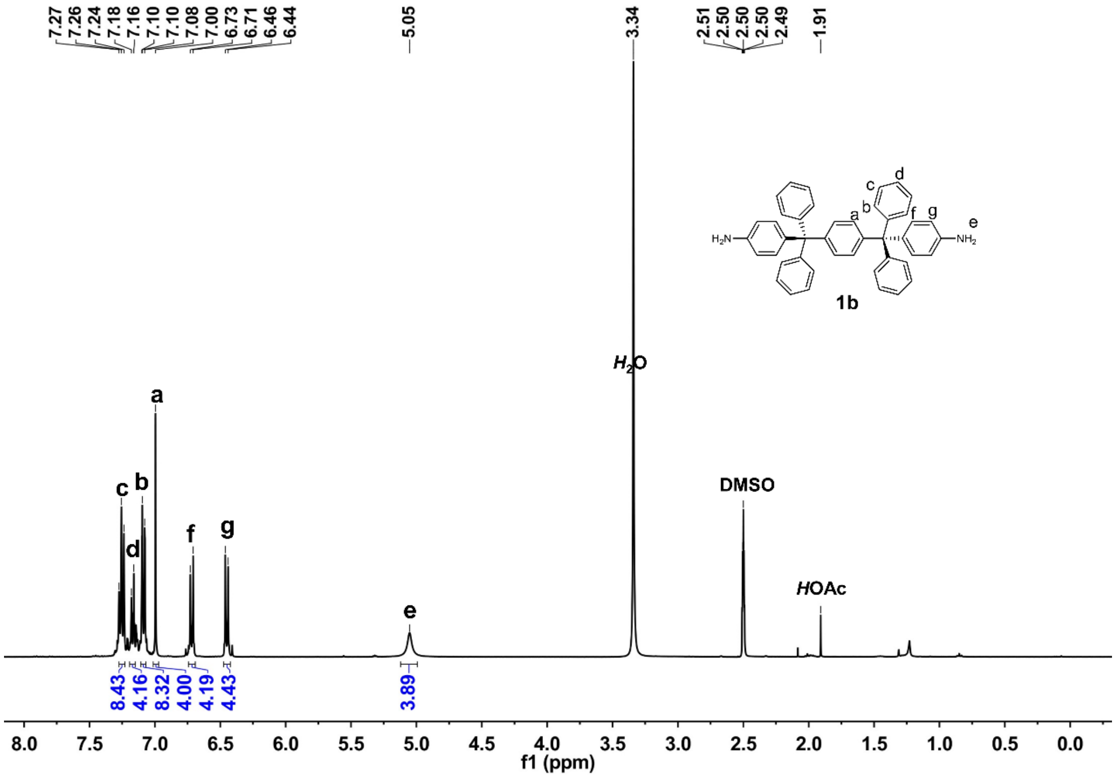

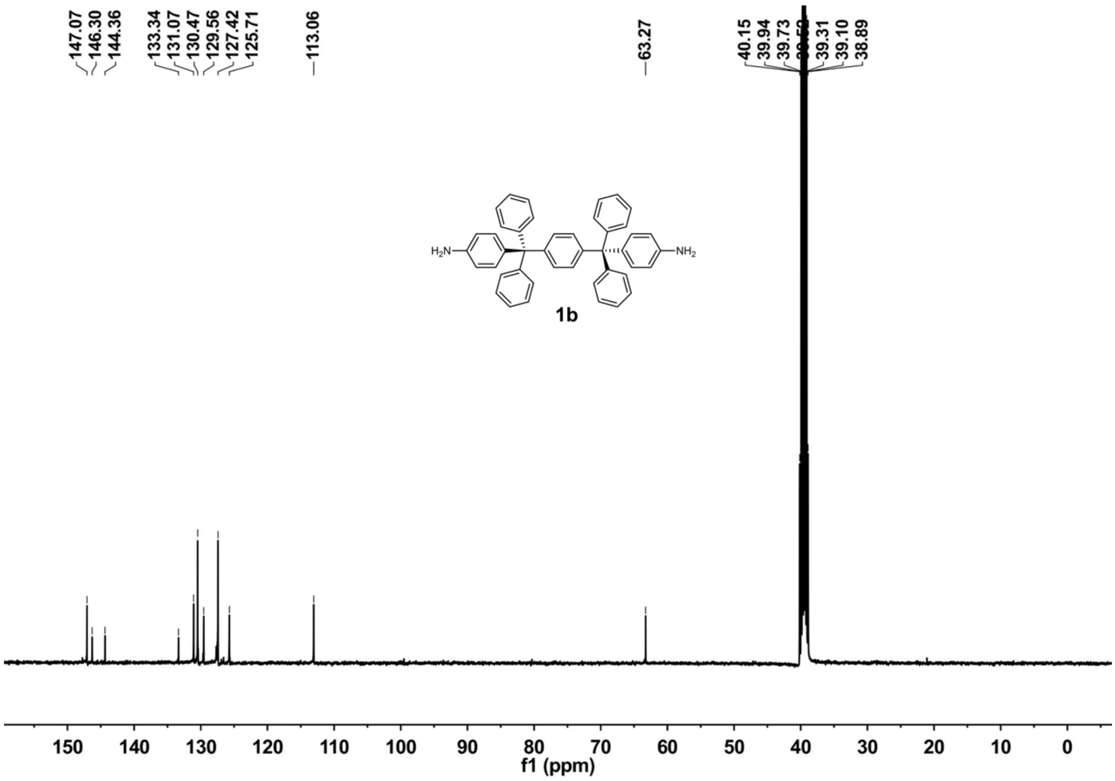

Supplementary Fig. 35 ^{1}H NMR (top, 400 MHz) and ^{13}C NMR (bottom, 101 MHz) spectra of 4,4'-(1,4-phenylenebis(diphenylmethylene))dianiline **1b** in DMSO.

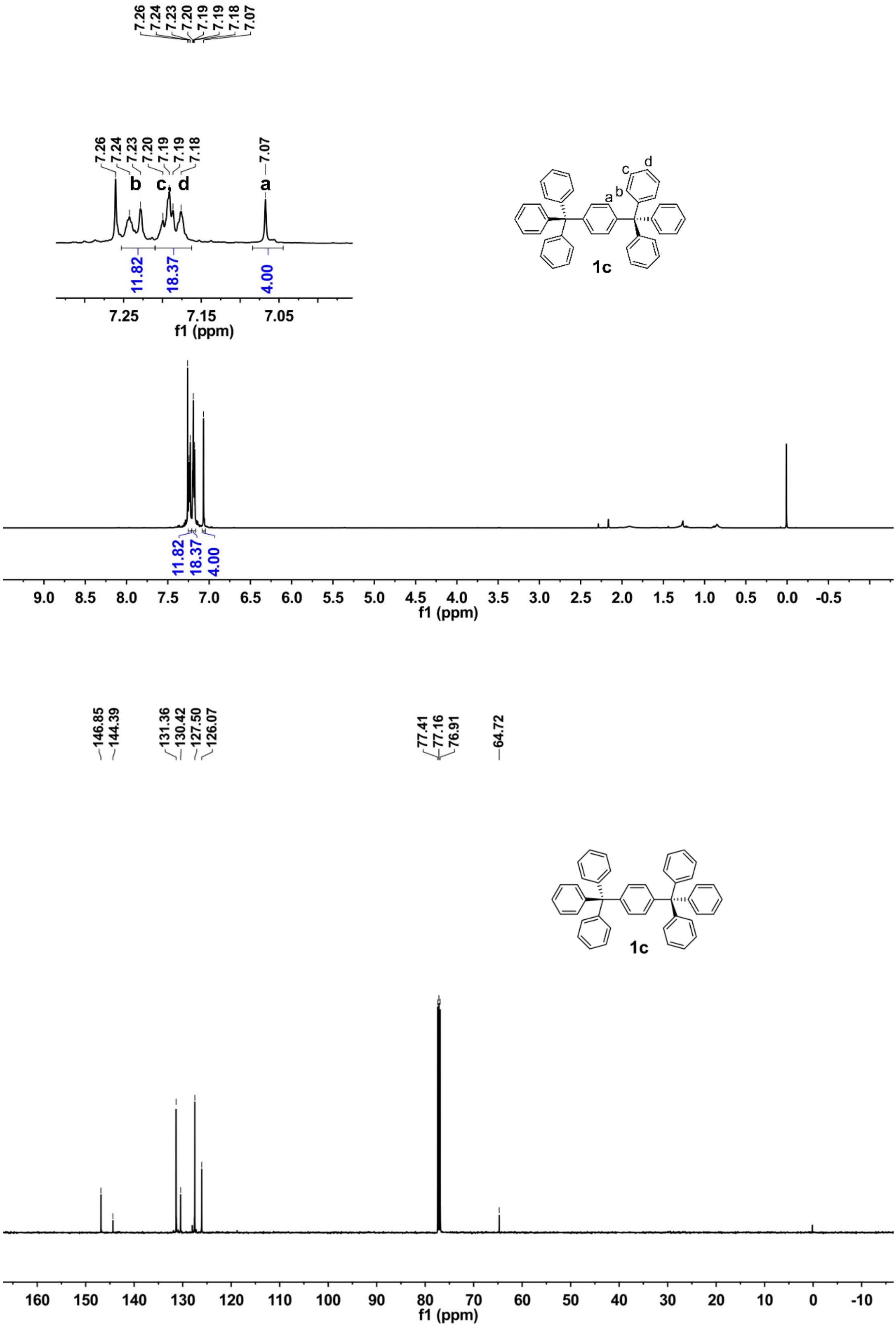

Supplementary Fig. 36 ^{1}H NMR (top, 400 MHz) and ^{13}C NMR (bottom, 101 MHz) spectra of 1,4-ditritylbenzene **1c** in $CDCl_3$.

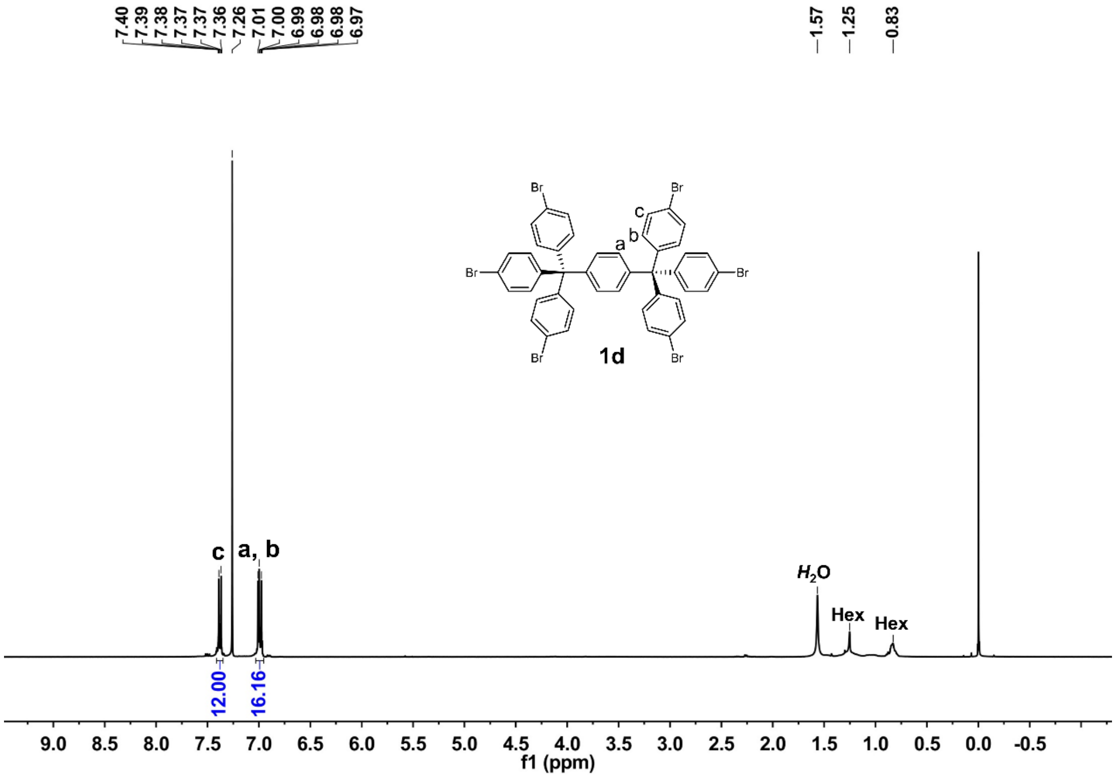

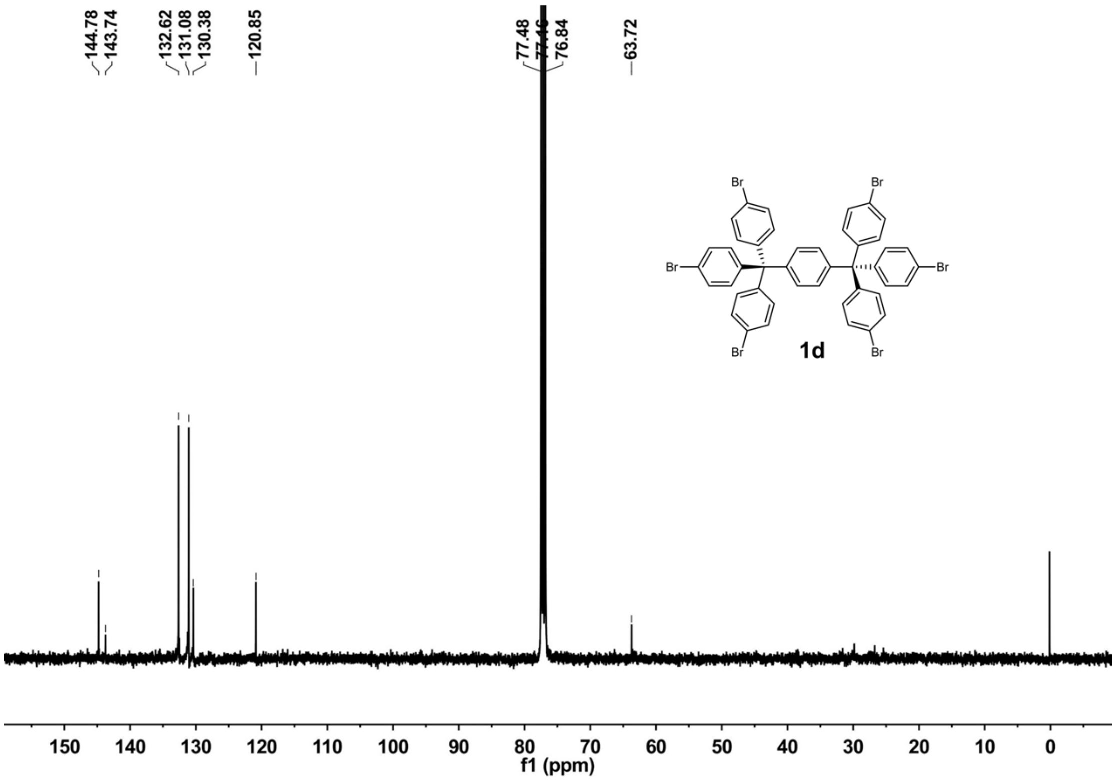

Supplementary Fig. 37 ^{1}H NMR (top, 400 MHz) and ^{13}C NMR (bottom, 101 MHz) spectra of 1,4-bis(tris(4-bromophenyl)methyl)benzene **1d** in $CDCl_3$.

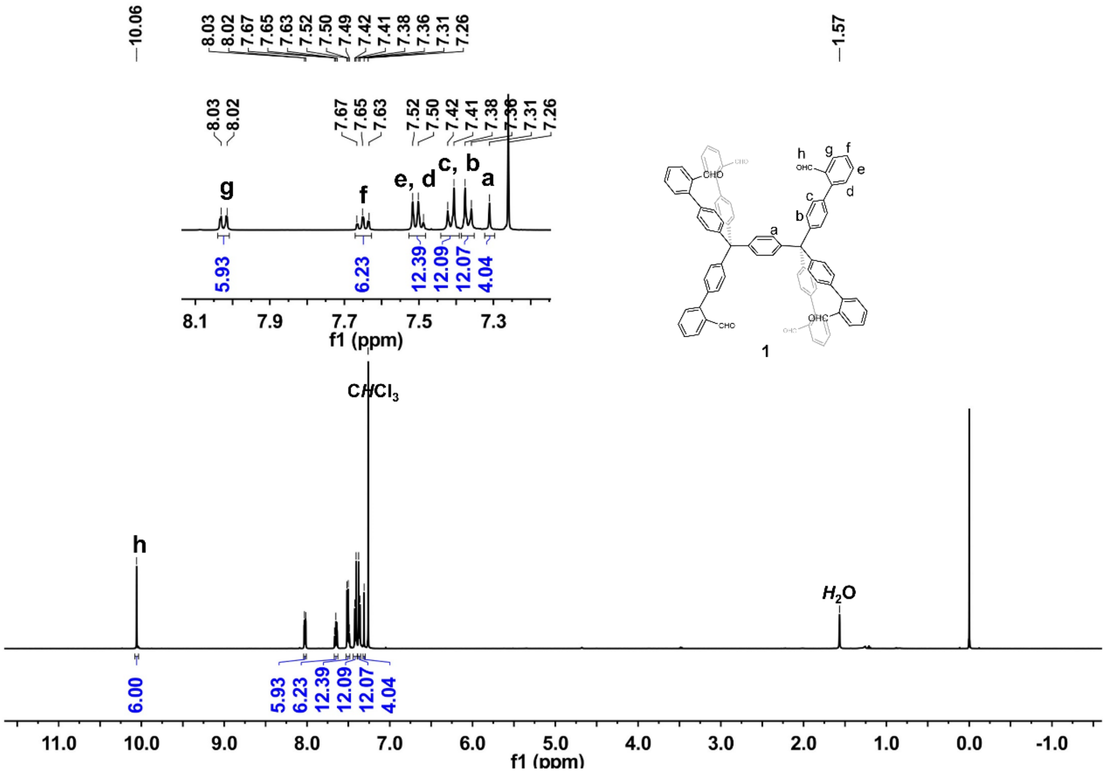

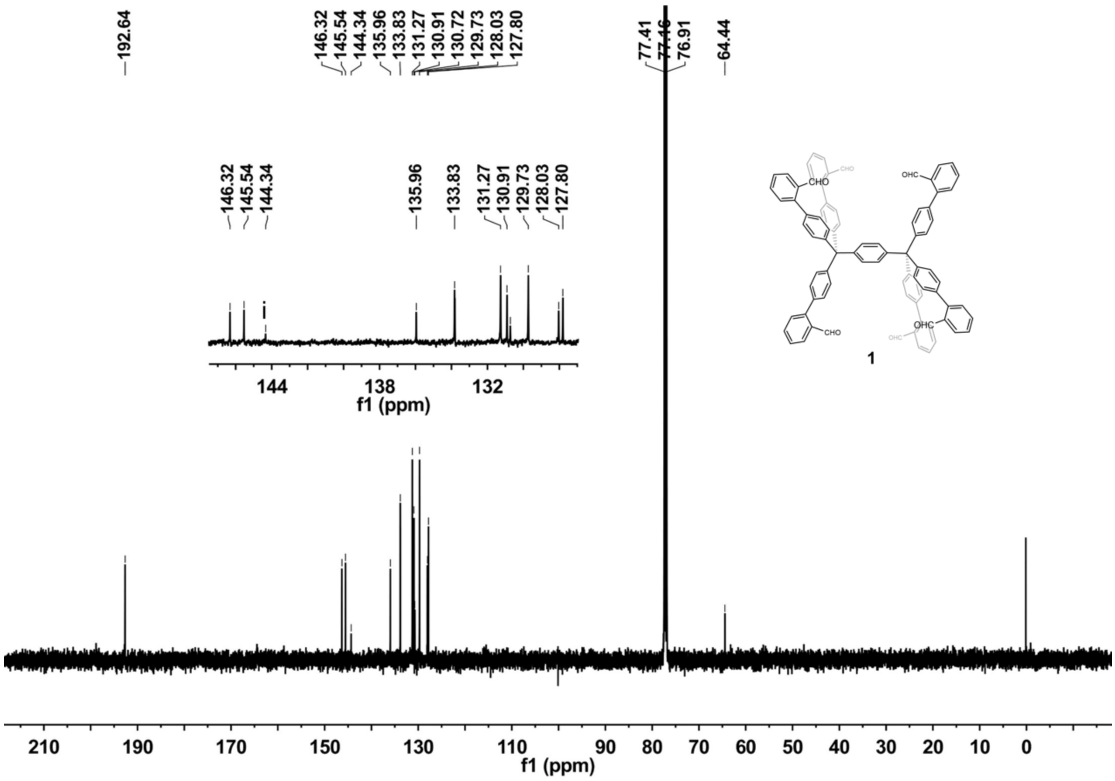

Supplementary Fig. 38 ^{1}H NMR (top, 400 MHz) and ^{13}C NMR (bottom, 101 MHz) spectra of model molecule **1** in $CDCl_3$.

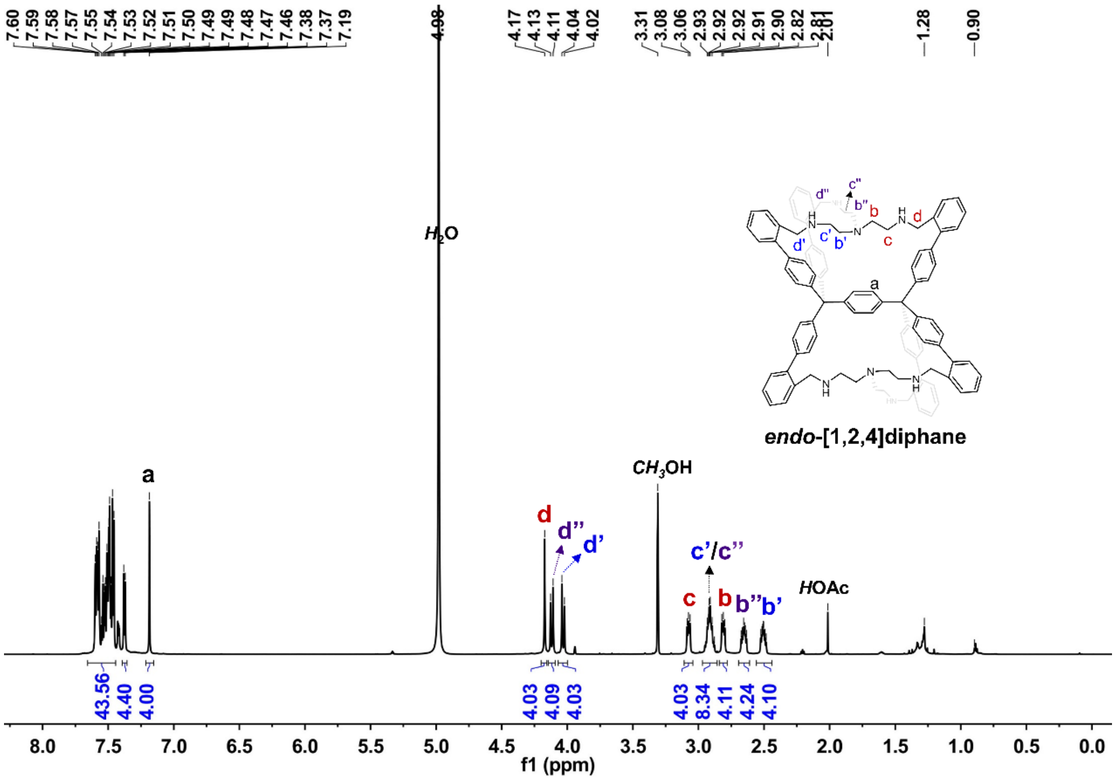

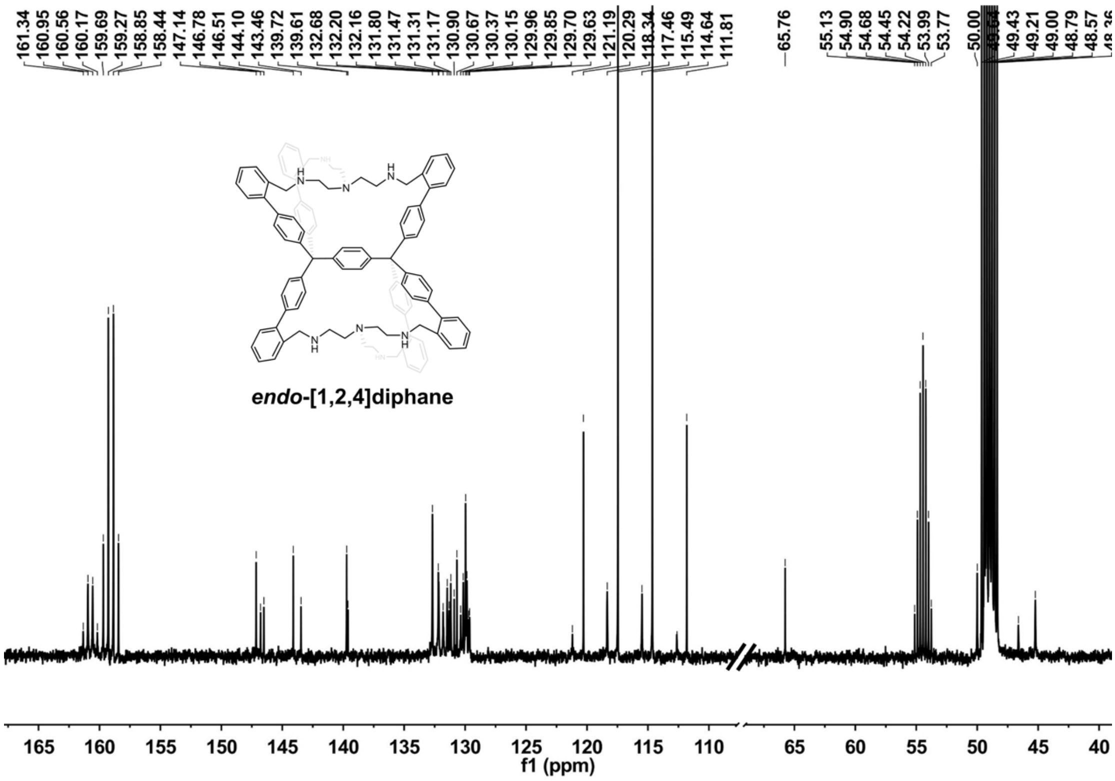

Supplementary Fig. 39 ^{1}H NMR (top, 400 MHz) and ^{13}C NMR (bottom, 101 MHz) spectra of *endo*-[1,2,4]diphane in CD_3OD.

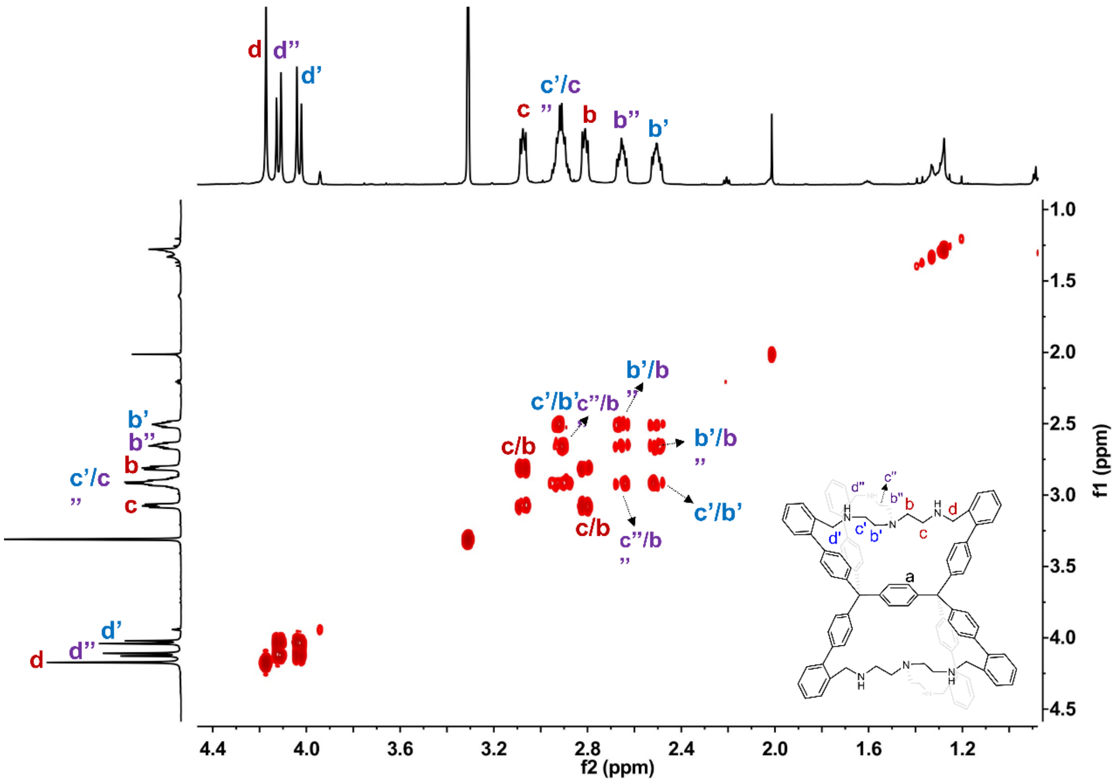

Supplementary Fig. 40 ^{1}H-^{1}H COSY spectrum (400 MHz) of *endo*-[1,2,4]diphane in CD_3OD.

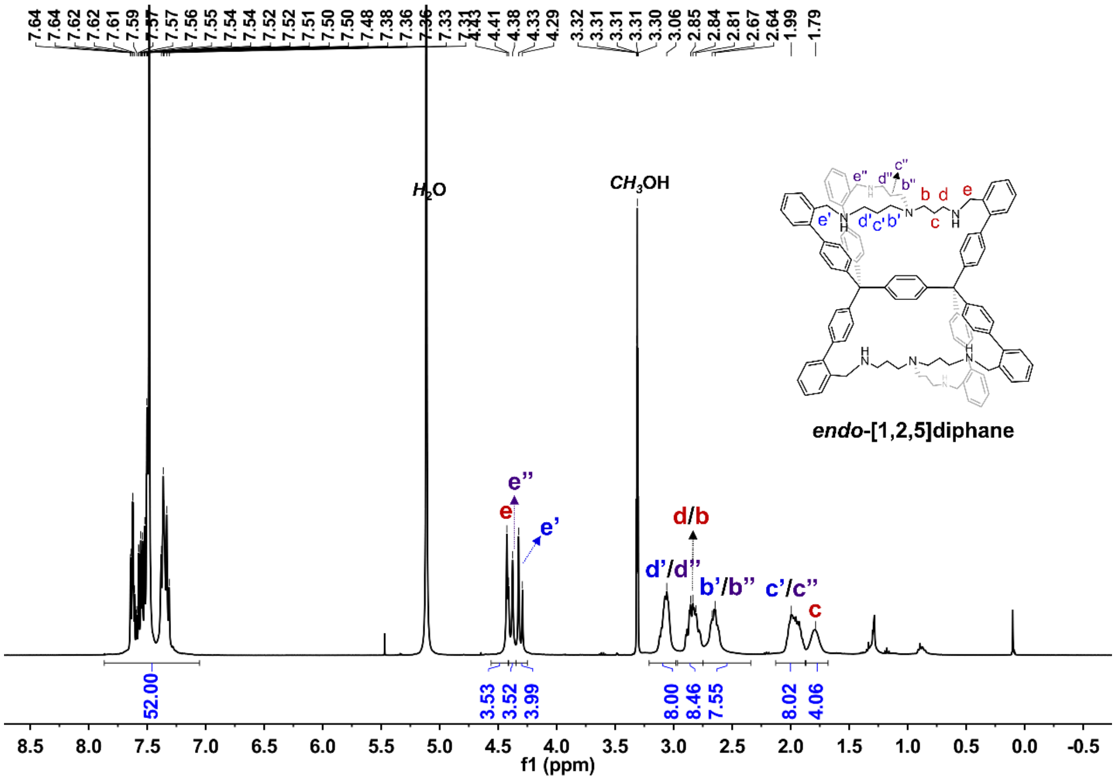

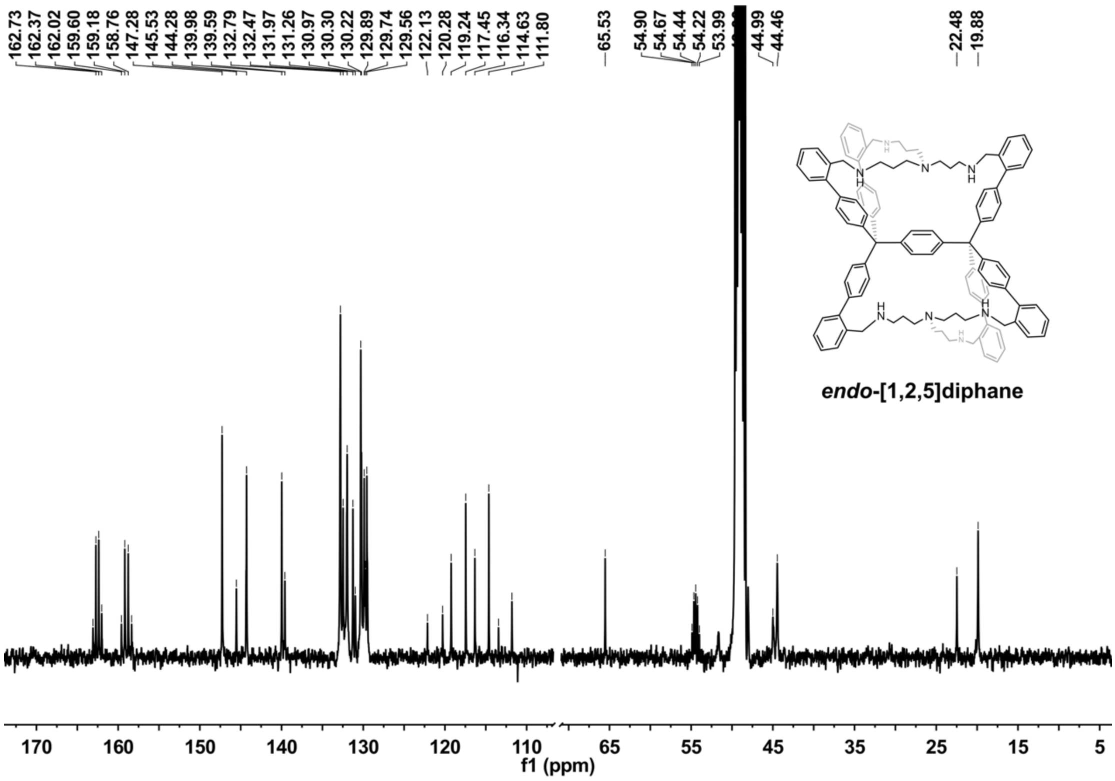

Supplementary Fig. 41 ^{1}H NMR (top, 400 MHz) and ^{13}C NMR (bottom, 101 MHz) spectra of *endo*-[1,2,5]diphane in CD_3OD.

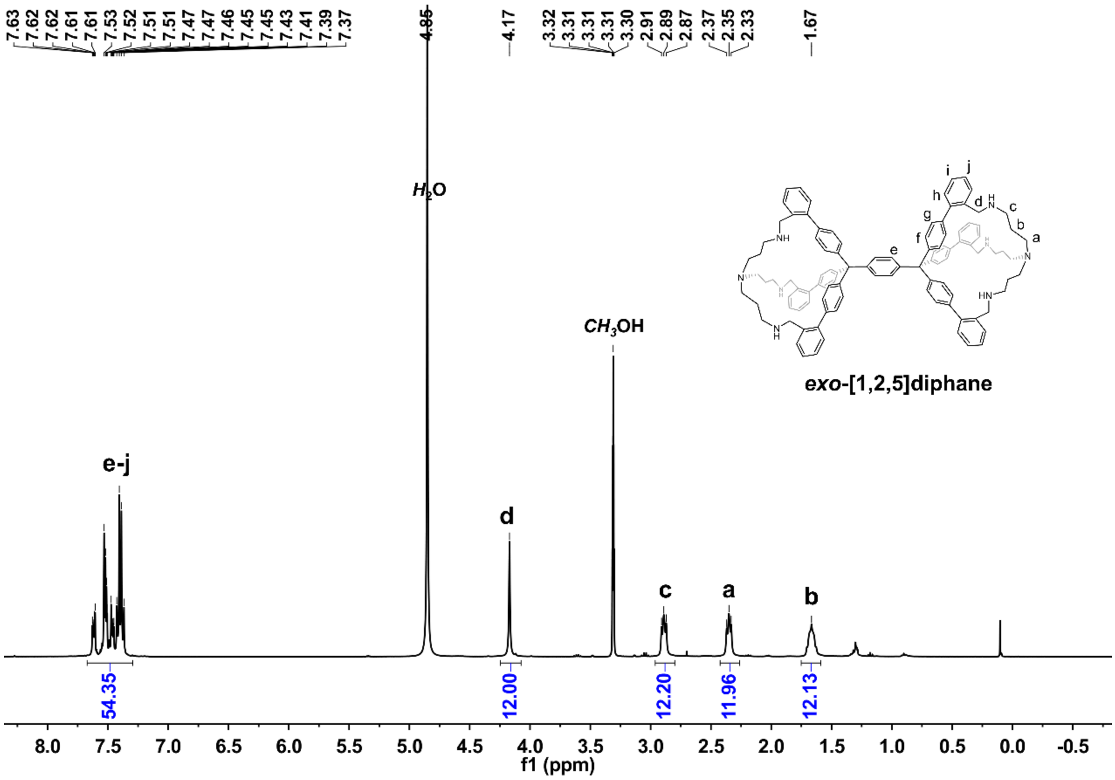

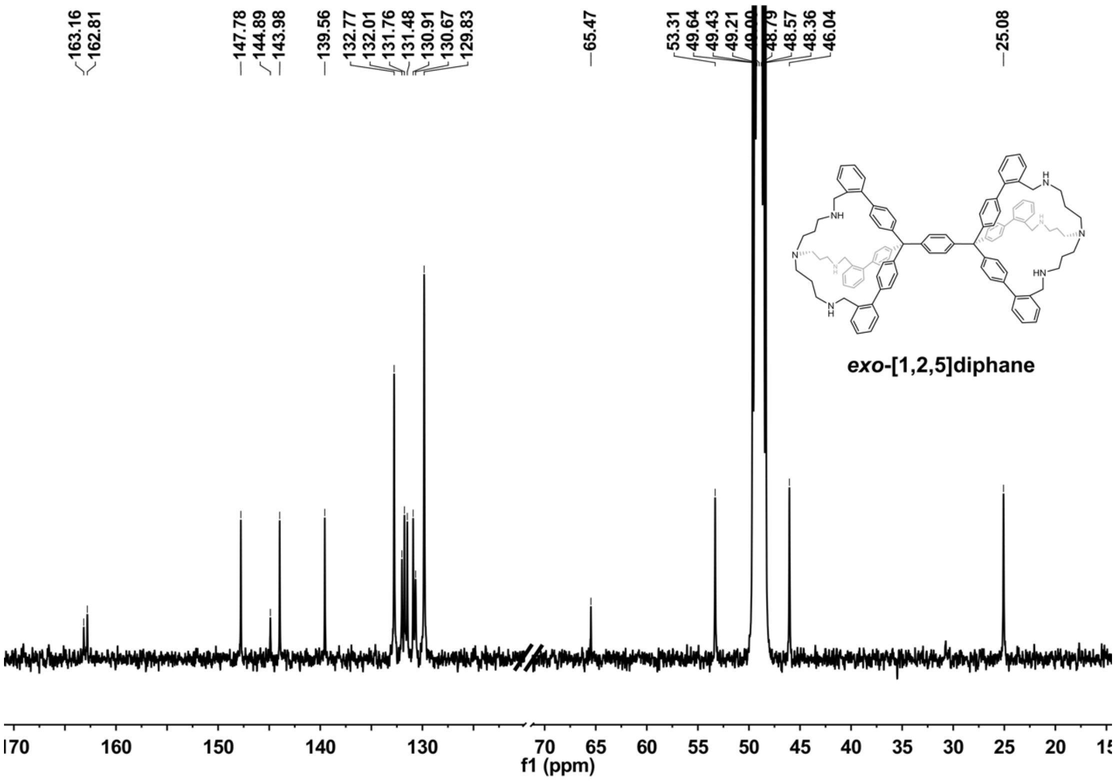

Supplementary Fig. 42 ^{1}H NMR (top, 400 MHz) and ^{13}C NMR (bottom, 101 MHz) spectra of *endo*-[1,2,5]diphane in CD_3OD.

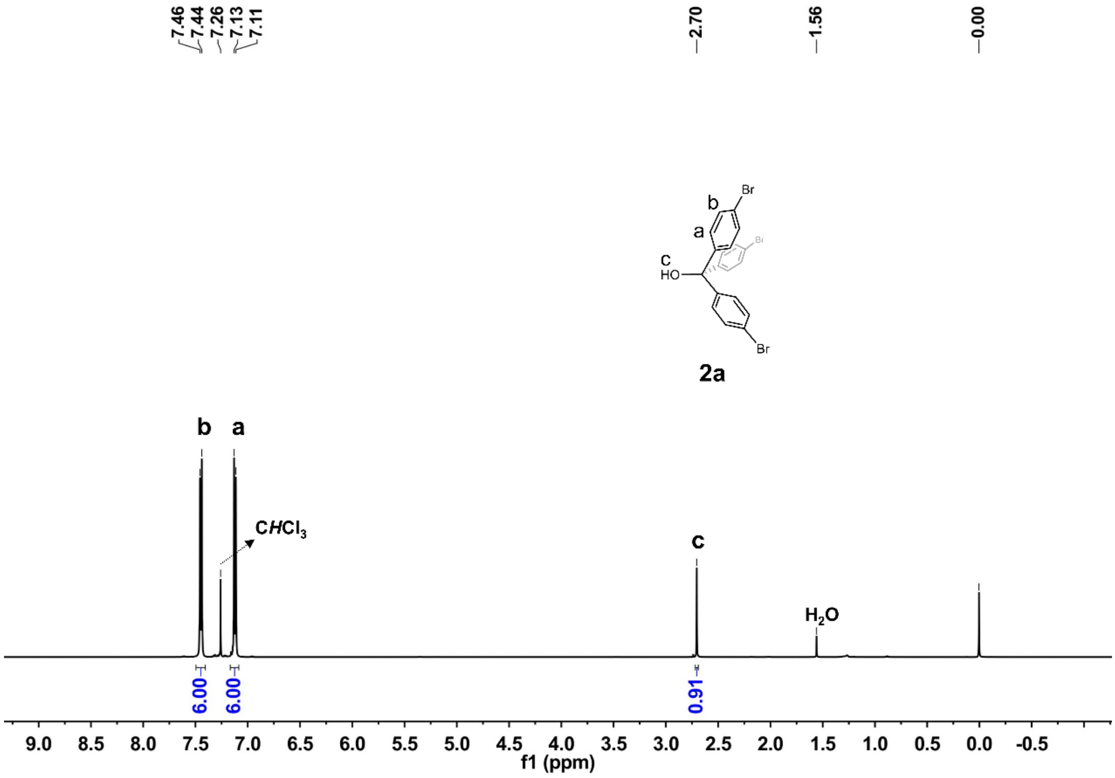

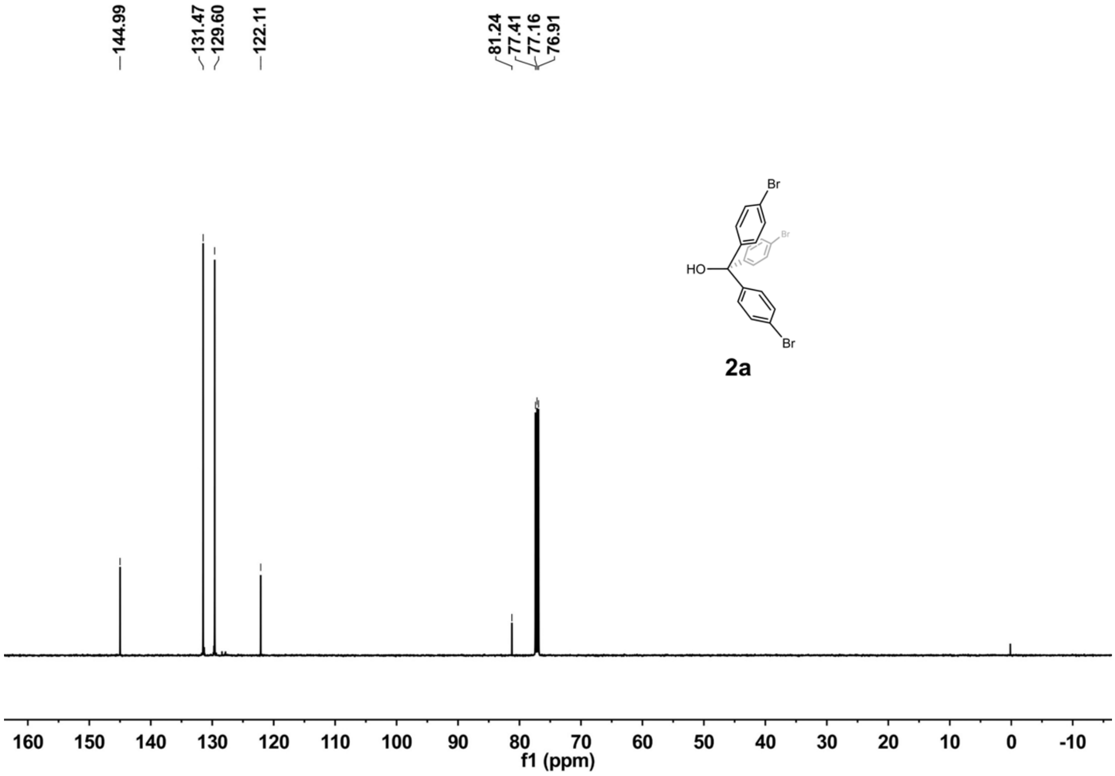

Supplementary Fig. 43 ^{1}H NMR (top, 400 MHz) and ^{13}C NMR (bottom, 101 MHz) spectra of tris(4-bromophenyl)methanol **2a** in $CDCl_3$.

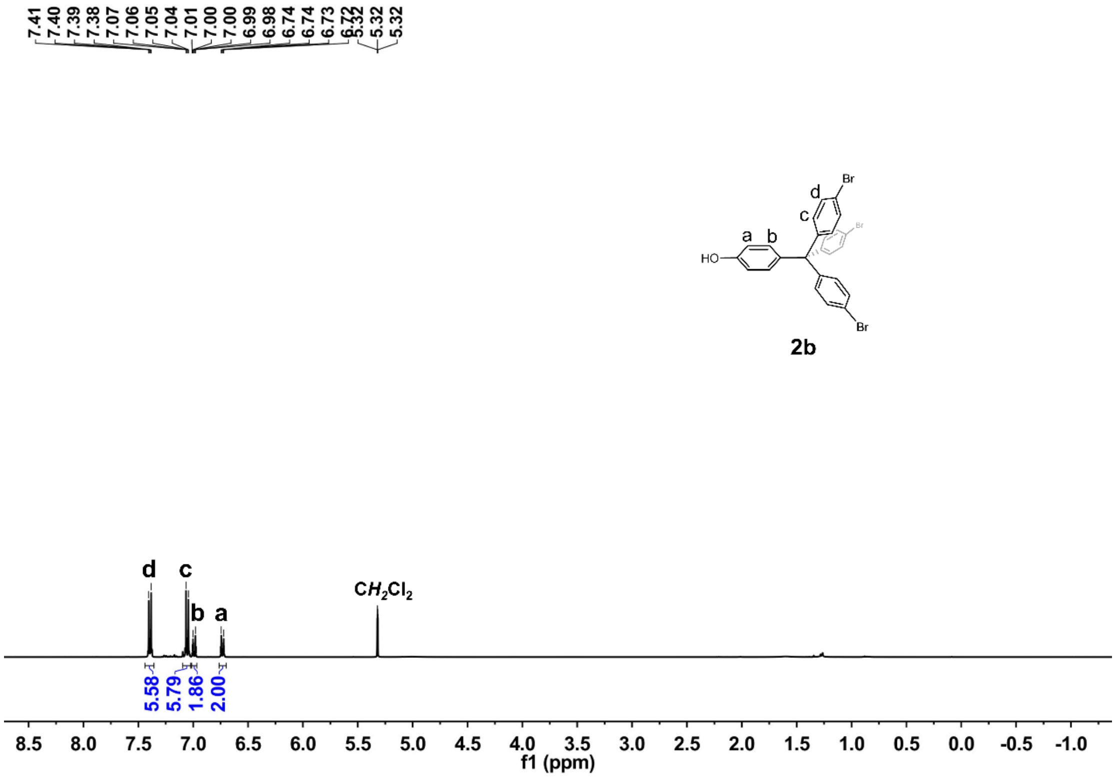

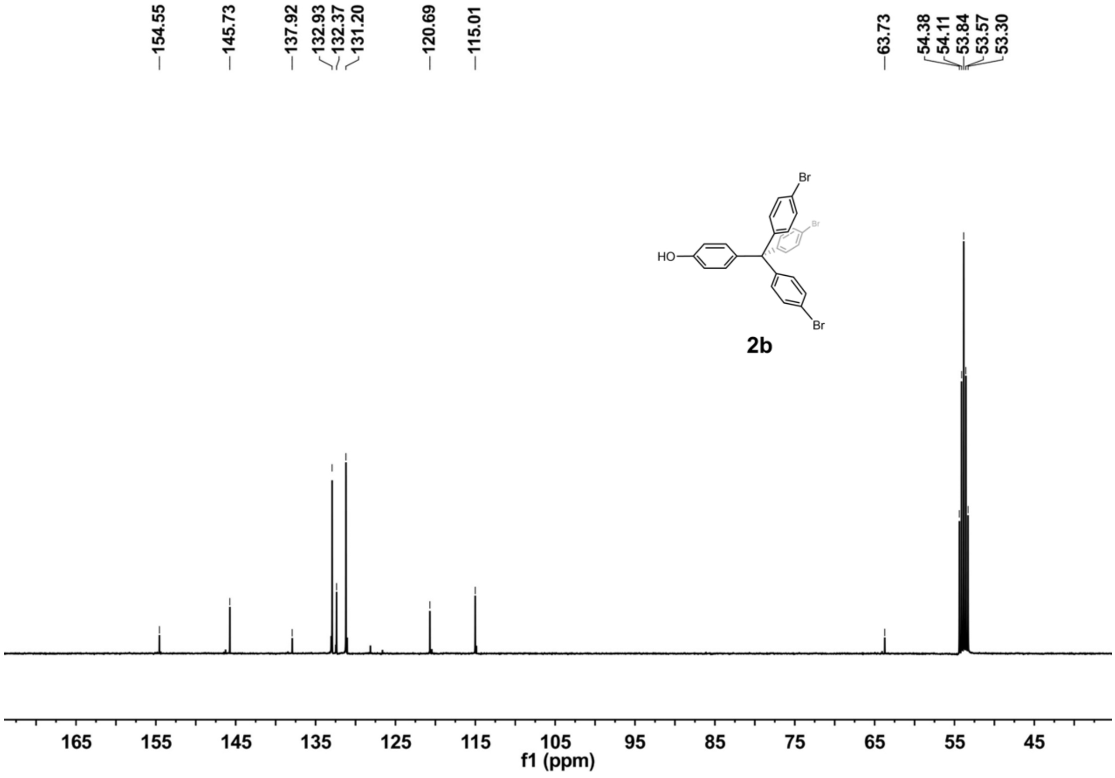

Supplementary Fig. 44 ^{1}H NMR (top, 400 MHz) and ^{13}C NMR (bottom, 101 MHz) spectra of 4-(tris(4-bromophenyl)methyl)phenol **2b** in $CDCl_3$.

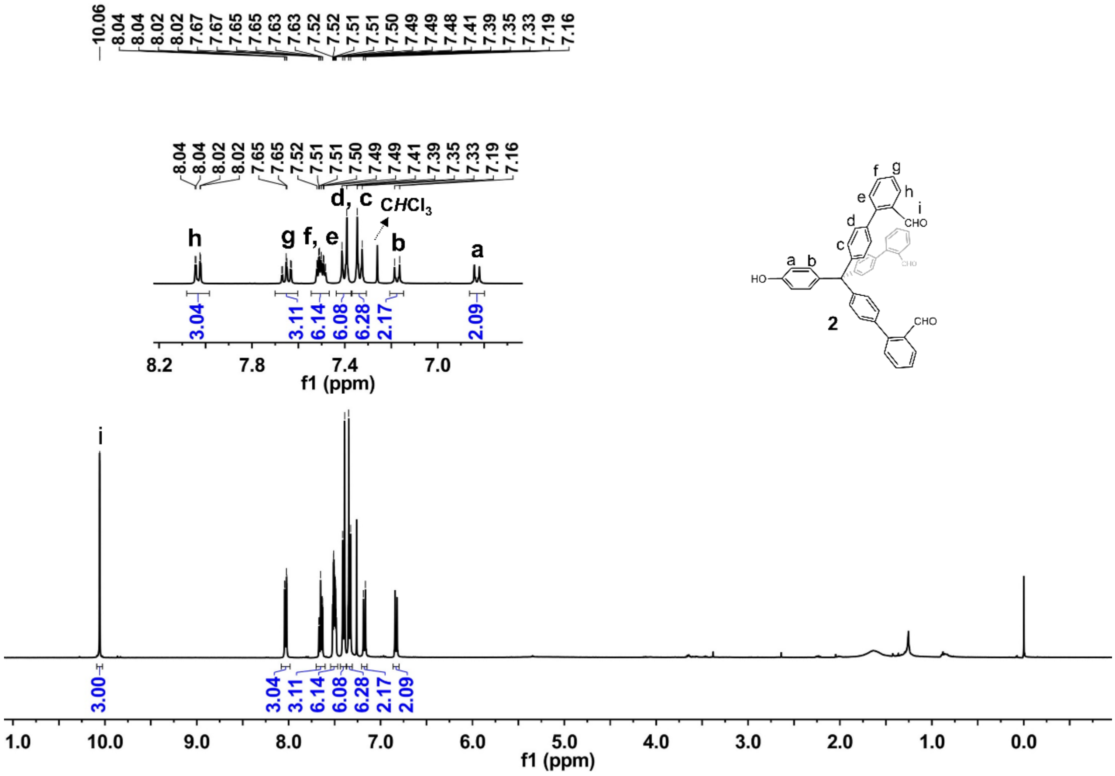

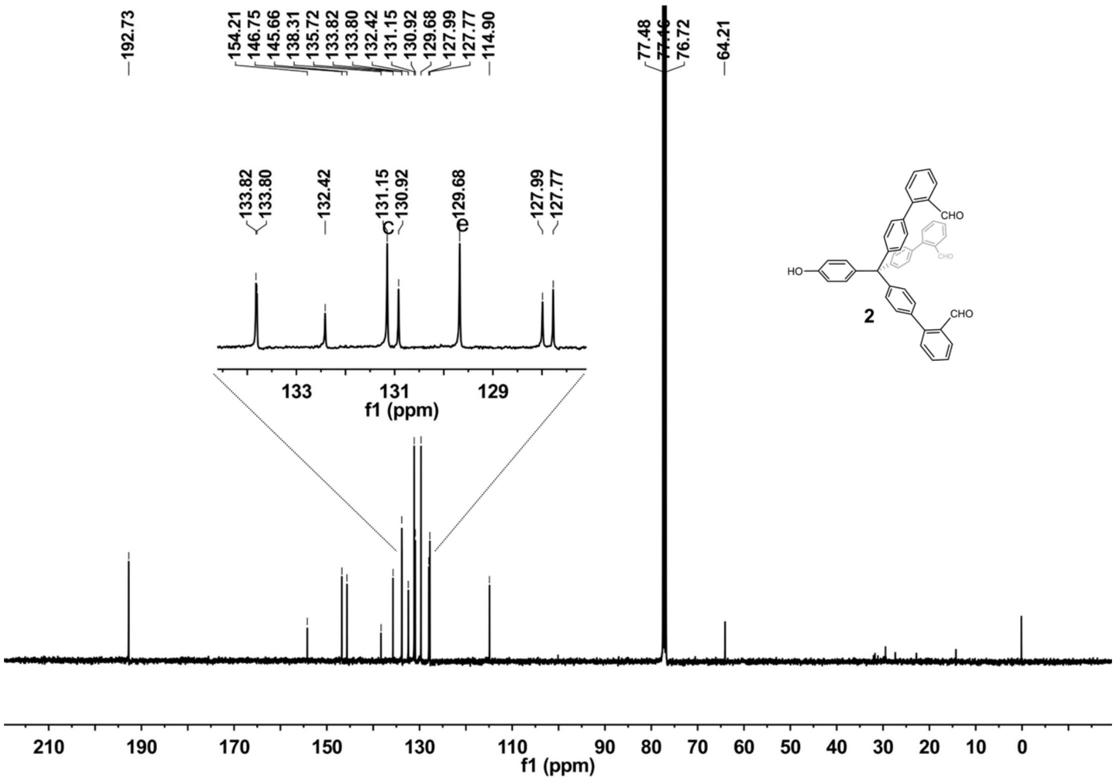

Supplementary Fig. 45 ^{1}H NMR (top, 400 MHz) and ^{13}C NMR (bottom, 101 MHz) spectra of 4-(tris(2'-formyl-[1,1'-biphenyl]-4-yl)methyl)phenol **2** in $CDCl_3$.

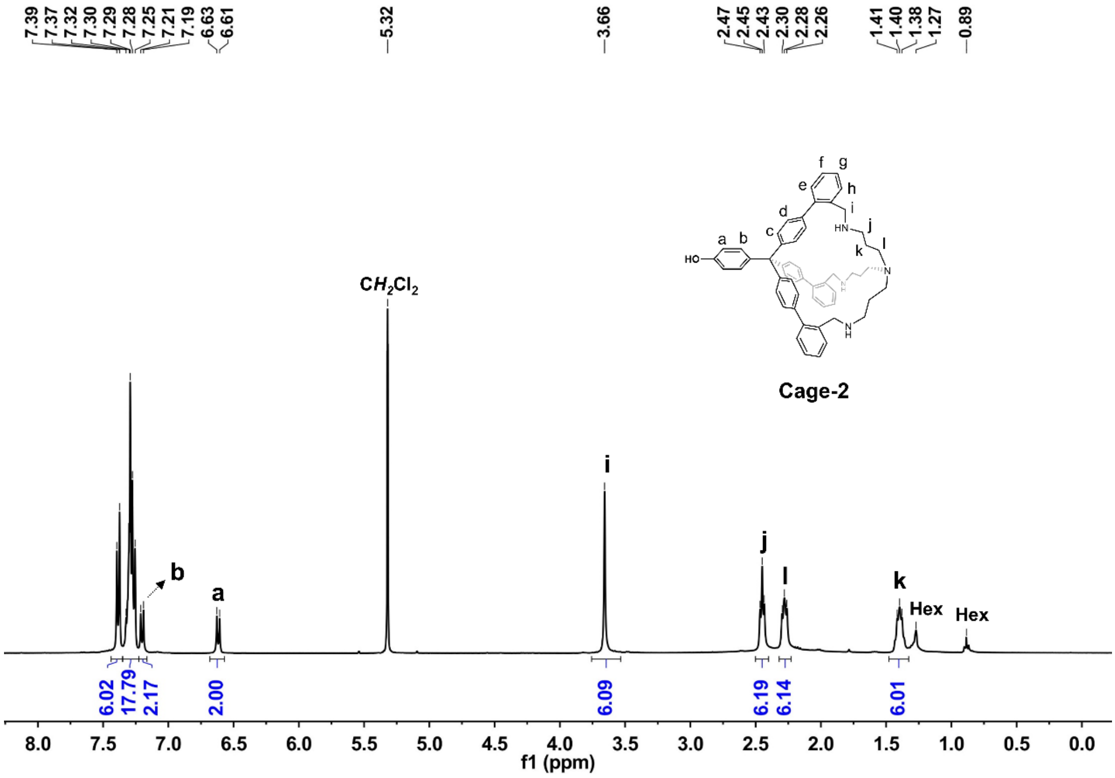

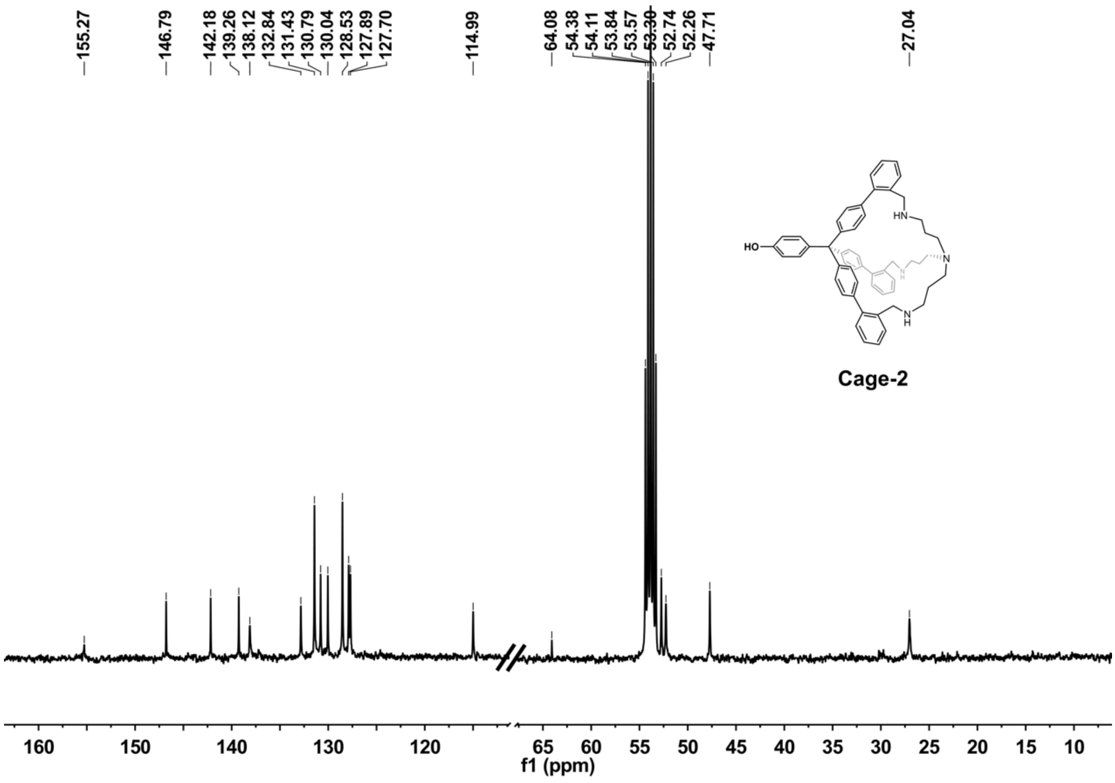

Supplementary Fig. 46 ^{1}H NMR (top, 400 MHz) and ^{13}C NMR (bottom, 101 MHz) spectra of Cage-2 in CD_2Cl_2.

12. Supplementary References

1. Plietzsch, O., Schade, A., Hafner, A., Huuskonen, J., Rissanen, K., Nieger, M., Muller, T.; Bräse, S. *Eur. J. Org. Chem.* **2013**, 283-299 (2013).

2. Schollmeyer, D., Shishkin, O. V., Ruhlc, T., Vysotsky, M. O. *CrystEngComm.* **10**, 715–723 (2008).

3. Gallina, M. E., Baytekin, B., Schalley, C., Ceroni, P. Light-Harvesting in Multichromophoric Rotaxanes. *Chem. Eur. J.* **18**, 1528–1535 (2012).

4. Dolomanov, O.V., Bourhis, L. J., Gildea, R. J, Howard, J. A. K. & Puschmann, H. *J. Appl. Cryst.* **42**, 339–341 (2009).

5. Sheldrick, G. M. *Acta Cryst. A* **71**, 3–8 (2015).

6. Sheldrick, G. M. *Acta Cryst. C* **71**, 3–8 (2015).

7. Frisch, M. J., Trucks, G. W., Schlegel, H. B., Scuseria, G. E., Robb, M. A., Cheeseman, J. R., Scalmani, G., Barone, V., Mennucci, B., Petersson, G. A., Nakatsuji, H., Caricato, M., Li, X., Hratchian, H. P., Izmaylov, A. F., Bloino, J., Zheng, G., Sonnenberg, J. L., Hada, M., Ehara, M., Toyota, K., Fukuda, R., Hasegawa, J., Ishida, M., Nakajima, T., Honda, Y., Kitao, O., Nakai, H., Vreven, T., Montgomery, J. A., Peralta, J. E., Ogliaro, F., Bearpark, M., Heyd, J. J., Brothers, E., Kudin, K. N., Staroverov, V. N., Keith, T., Kobayashi, R., Normand, J., Raghavachari, K., Rendell, A., Burant, J. C., Iyengar, S. S., Tomasi, J., Cossi, M., Rega, N., Millam, J. M., Klene, M., Knox, J. E., Cross, J. B., Bakken, V., Adamo, C., Jaramillo, J., Gomperts, R., Stratmann, R. E., Yazyev, O., Austin, A. J., Cammi, R., Pomelli, C., Ochterski, J. W., Martin, R. L., Morokuma, K., Zakrzewski, V. G., Voth, G. A., Salvador, P., Dannenberg, J. J., Dapprich, S., Daniels, A. D., Farkas, O., Foresman, J. B., Ortiz, J. V., Cioslowski, J., Fox, D. J. *Gaussian 09*, revision D. 01; Gaussian, Inc. Wallingford CT, (2013).

8. Vosko, S., Wilk, L., Nusair, M. *Can. J. Phys.* **58**, 1200–1211 (1980).

9. Becke, A. D. *J. Chem. Phys.* **107**, 8554–8560 (1997).

10. Kohn, W., Becke, A. D., Parr, R. G. *J. Phys. Chem.* **100**, 12974–12980 (1996).

11. Adamo, C., Barone, V. *J. Chem. Phys.* **110**, 6158–6170 (1999).

12. Grimme, S., Antony, J., Ehrlich, S., Krieg, H. *J. Chem. Phys.* **132**, 154104 (2010).

13. Francl, M. M., Pietro, W. J., Hehre, W. J., Binkley, J. S., Gordon, M. S., Defrees, D. J., Pople, J. A. *J. Chem. Phys.* **77**, 3654–3665 (1982).

14. Goerigkab, L and Grimme, S. Phys. *Chem. Chem. Phys.* **13**, 6670–6688 (2011).

15. Zhao, Y., Truhlar, D. G. *Theor. Chem. Acc.* **120**, 215–41 (2008).

16. Wang, J., Wang, W., Kollman, P. A., Case, D. A. *J. Mol. Graph. Model.* **25**, 247–260 (2006).

17. Wang, J., Wolf, R. M., Caldwell, J. W., Kollman, P. A., Case, D. A. *J. Comput. Chem.* **25**, 1157–1174 (2004).

18. Berendsen, H. J. C., van der Spoel, D., van Drunen, R. *Comp. Phys. Comm.* **91**, 43–56 (1995).

19. Abraham, M. J., Murtola, T., Schulz, R., Páll, S., Smith, J. C., Hess, B., Lindahl, E. *SoftwareX* **1-2***,* 19–25 (2015).

20. da Silva, A. W. S., Vranken, W. F. *BMC Research Notes* **5**, 367 (2012).

21. Pascal, T. A., Lin, S. T., Goddard, W. A., III. *Phys. Chem. Chem. Phys.* **13**, 169–181 (2011).

22. Bussi, G., Donadio, D., Parrinello, M. *J. Chem. Phys.* **126**, 014101 (2007).

23. Salva, A., Donoso, J., Frau, J., Munoz, F. *J. Phys. Chem. A*. **107**, 9409–9414 (2003).
